# Anoxygenic Phototrophs Span Geochemical Gradients and Diverse Morphologies in Terrestrial Geothermal Springs

**DOI:** 10.1128/mSystems.00498-19

**Published:** 2019-11-05

**Authors:** Trinity L. Hamilton, Annastacia C. Bennett, Senthil K. Murugapiran, Jeff R. Havig

**Affiliations:** a Department of Plant and Microbial Biology, University of Minnesota, St. Paul, Minnesota, USA; b BioTechnology Institute, University of Minnesota, St. Paul, Minnesota, USA; c Department of Earth and Environmental Sciences, University of Minnesota, Minneapolis, Minnesota, USA; California Department of Water Resources

**Keywords:** hot springs, photoassimilation, phototroph, anoxygenic photosynthesis, *Chloroflexi*, sulfide, aerobic anoxygenic phototroph, pH, temperature, *Cyanobacteria*, *Chlorobi*, Yellowstone National Park, anoxygenic

## Abstract

Extant anoxygenic phototrophs are taxonomically, physiologically, and metabolically diverse and include examples from all seven bacterial phyla with characterized phototrophic members. pH, temperature, and sulfide are known to constrain phototrophs, but how these factors dictate the distribution and activity of specific taxa of anoxygenic phototrophs has not been reported. Here, we hypothesized that within the known limits of pH, temperature, and sulfide, the distribution, abundance, and activity of specific anoxygenic phototrophic taxa would vary due to key differences in the physiology of these organisms. To test this hypothesis, we examined the distribution, abundance, and potential activity of anoxygenic phototrophs in filaments, microbial mats, and sediments across geochemical gradients in geothermal features of Yellowstone National Park, which ranged in pH from 2.2 to 9.4 and in temperature from 31.5°C to 71.0°C. Indeed, our data indicate putative aerobic anoxygenic phototrophs within the *Proteobacteria* are more abundant at lower pH and lower temperature, while phototrophic *Chloroflexi* are prevalent in circumneutral to alkaline springs. In contrast to previous studies, our data suggest sulfide is not a key determinant of anoxygenic phototrophic taxa. Finally, our data underscore a role for photoheterotrophy (or photomixotrophy) across geochemical gradients in terrestrial geothermal ecosystems.

**IMPORTANCE** There is a long and rich history of literature on phototrophs in terrestrial geothermal springs. These studies have revealed sulfide, pH, and temperature are the main constraints on phototrophy. However, the taxonomic and physiological diversity of anoxygenic phototrophs suggests that, within these constraints, specific geochemical parameters determine the distribution and activity of individual anoxygenic phototrophic taxa. Here, we report the recovery of sequences affiliated with characterized anoxygenic phototrophs in sites that range in pH from 2 to 9 and in temperature from 31°C to 71°C. Transcript abundance indicates anoxygenic phototrophs are active across this temperature and pH range. Our data suggest sulfide is not a key determinant of anoxygenic phototrophic taxa and underscore a role for photoheterotrophy in terrestrial geothermal ecosystems. These data provide the framework for high-resolution sequencing and *in situ* activity approaches to characterize the physiology of specific anoxygenic phototrophic taxa across a broad range of temperatures and pH.

## Abstract (precis)

There is a long and rich history of literature on phototrophs in terrestrial geothermal springs. These studies have revealed sulfide, pH, and temperature are the main constraints on phototrophy. However, the taxonomic and physiological diversity of anoxygenic phototrophs suggests that, within these constraints, specific geochemical parameters determine the distribution and activity of individual anoxygenic phototrophic taxa. Here, we report the recovery of sequences affiliated with characterized anoxygenic phototrophs in sites that range in pH from 2 to 9 and in temperature from 31°C to 71°C. Transcript abundance indicates anoxygenic phototrophs are active across this temperature and pH range. Our data suggest sulfide is not a key determinant of anoxygenic phototrophic taxa and underscore a role for photoheterotrophy in terrestrial geothermal ecosystems. These data provide the framework for high-resolution sequencing and *in situ* activity approaches to characterize the physiology of specific anoxygenic phototrophic taxa across a broad range of temperatures and pH.

## INTRODUCTION

Anoxygenic phototrophs are taxonomically and metabolically diverse. At least seven bacterial phyla contain members that are at least facultative anoxygenic phototrophs (1). Anoxygenic phototrophs are observed in a wide range of habitats, including euxinic lakes, microbial mats, hot springs, and hypersaline lagoons, where they often exist in close proximity to oxygenic phototrophs. The ability to harvest light and tolerance to oxygen are most often cited as the key factors governing the occurrence of phototrophs in ecological niches, with implications for the evolution of photosynthesis during the Archean and Paleoproterozoic eras (2). At a broader scale, studies of phototrophs in terrestrial hot springs have identified temperature, pH, and sulfide as the key determinants of the distribution of both oxygenic and anoxygenic phototrophs (3–5). These studies have relied on tools that lack the fine-scale taxonomic resolution necessary to delineate the full diversity of anoxygenic phototrophs (e.g., pigments, visual evidence of pigmented biomass, and detection of marker genes). In contrast, in-depth single-site studies have resolved distinct species (or ecotypes) of phototrophs and their physiology (reviewed in reference 6). The differences in metabolism and physiology between phototrophic taxa suggest temperature, pH, and sulfide affect the distribution and activity of individual taxa. Indeed, phototrophic algae are predominant in low-pH hot springs (pH <5.0), while *Cyanobacteria* are abundant in alkaline hot springs (7–9). However, analogous observations for the predominance of specific anoxygenic phototrophic taxa have not been reported.

Phototrophs use photochemical reaction centers containing (bacterio)chlorophylls (BChls or Chls) to capture solar energy and convert it into chemical energy (10). Anoxygenic phototrophs are distinct from oxygenic phototrophs in that they employ one reaction center, either type 1 (RCI) or type 2 (RCII), and generate energy and reducing power through the oxidation of compounds including organic molecules, Fe^2+^, H_2_, S^0^, HS^−^, S_2_O_3_^2−^, NO_2_^−^, and AsO_3_^3−^ (11). Anoxygenic phototrophs can be photoautotrophic, using light for energy and reducing power to fix inorganic carbon. They can also be photoheterotrophic or photomixotrophic. Carbon fixation pathways observed in autotrophic anoxygenic phototrophs include the rTCA cycle (reverse tricarboxylic acid cycle), the CBB cycle (Calvin-Benson-Bassham cycle), and the 3HPB cycle (3-hydroxypropionate bi-cycle) (12). Phototrophic members of the *Firmicutes*, *Acidobacteria*, *Heliobacteria*, and *Gemmatimonadetes* described to date do not fix carbon, whereas the *Proteobacteria*, *Chloroflexi*, and *Chlorobi* include examples of photoautotrophs, photoheterotrophs, or photomixotrophs (12–14). Some anoxygenic phototrophs display a range of oxygen tolerance while others are strict anaerobes, and some anoxygenic phototrophs are also capable of aerobic chemoheterotrophic growth (15–17). Two particular phyla with putative phototrophs embody the range of diversity of anoxygenic phototrophy: the *Proteobacteria* and the *Chloroflexi*. *Proteobacteria* include examples of aerobic anoxygenic phototrophs in the *Alpha*- and *Gammaproteobacteria* (13, 15–17). *Chloroflexi* observed in hot springs have shown variation in carbon assimilation strategies (chemoorganotrophy versus photoheterotrophy versus photoautotrophy) across a diurnal cycle (18).

There is a long and rich history of literature on phototrophs in terrestrial geothermal springs where reduced substrates, e.g., hydrogen, hydrogen sulfide, and sometimes ferrous iron, to support anoxygenic phototrophs, are typically abundant. Sequences affiliated with anoxygenic phototrophs have been recovered from terrestrial hot springs in Yellowstone National Park (YNP) (5, 6, 19–28). Anoxygenic phototrophs have also been observed in hot springs around the world, including the Arctic (29), New Zealand (30), Japan (reviewed in reference 31), Iceland (32), Russia (33), Malaysia (34), China (35), the Tibetan plateau (36), Oregon, USA (37), and Nevada, USA (38).

Previous studies in YNP have reported intensive studies of phototrophic mats at a single site (e.g., Mushroom and Octopus Springs), revealing fine-scale interactions in phototrophic mats and ecological diversification in cyanobacteria (reviewed in reference 6). Others have employed tools that lack the fine-scale taxonomic resolution necessary to delineate the full diversity of anoxygenic phototrophs, such as reporting phototrophs by visual observations of pigmented biomass (e.g., see reference 4), the presence of pigments (e.g., see references 5, 39, and 40), and positive amplification of bacteriochlorophyll biosynthesis genes (e.g., *bchY* and *chlL/bchL*). *bchY* encodes a component of chlorophyllide (Chlide) oxidoreductase, which reduces the B-ring of Chlide *a* (41), and has been employed as a proxy to characterize the distribution and diversity of anoxygenic phototrophs (*Proteobacteria*, *Chlorobi*, *Chloroflexi*, *Acidobacteria*, and *Firmicutes*) (5, 42, 43). All known bacterial phototrophs carry *chlL/bchL* (41, 44–46), which encodes a component of the enzyme complex that catalyzes the light-independent stereospecific reduction in the D-ring of protochlorophyllide *a*, resulting in Chlide *a* (47–49).

Here, we examined the distribution, abundance, and potential activity of putative anoxygenic phototrophs across geochemical gradients in geothermal features of YNP, ranging in pH from 2.2 to 9.4 and in temperature from 31.5°C to 71.0°C. Within this framework, we hypothesized that biotic and abiotic factors, including pH, temperature, and sulfide, would select for specific anoxygenic phototrophic taxa due to key differences in the physiology of these organisms.

## RESULTS AND DISCUSSION

Terrestrial geothermal springs provide an exceptional opportunity to study diverse microbes, including phototrophs, and their potential metabolisms across geochemical gradients (e.g., see references 50–52). Previous studies indicate temperature, pH, and sulfide are the main determinants of the distribution of phototrophs in YNP and that anoxygenic phototrophy is more constrained by temperature and pH than oxygenic phototrophs (3–5, 53). However, these studies employed broad markers of phototrophs (presence of pigments or [bacterio]chlorophyll biosynthesis genes) and were not able to resolve specific anoxygenic phototrophic taxa that vary taxonomically and physiologically. We hypothesized that the physiological differences between anoxygenic phototrophs, temperature, pH, and sulfide would affect the distribution of individual taxa of anoxygenic phototrophs within the observed limits of phototrophy. To assess this, we employed quantitative reverse transcription-PCR (qRT-PCR) and *in situ* microcosms to inform the range of temperature, pH, and sulfide concentration where anoxygenic phototrophs are abundant and active. We used 16S rRNA gene amplicon sequencing and stable isotopes of C and N to identify specific taxa of putative anoxygenic phototrophs and their potential metabolism within this temperature and pH range. From these data, we assessed biotic and abiotic factors that affect the distribution of specific anoxygenic phototrophic taxa.

### Occurrence, abundance, and potential activity of anoxygenic phototrophs.

**FIG 1:**
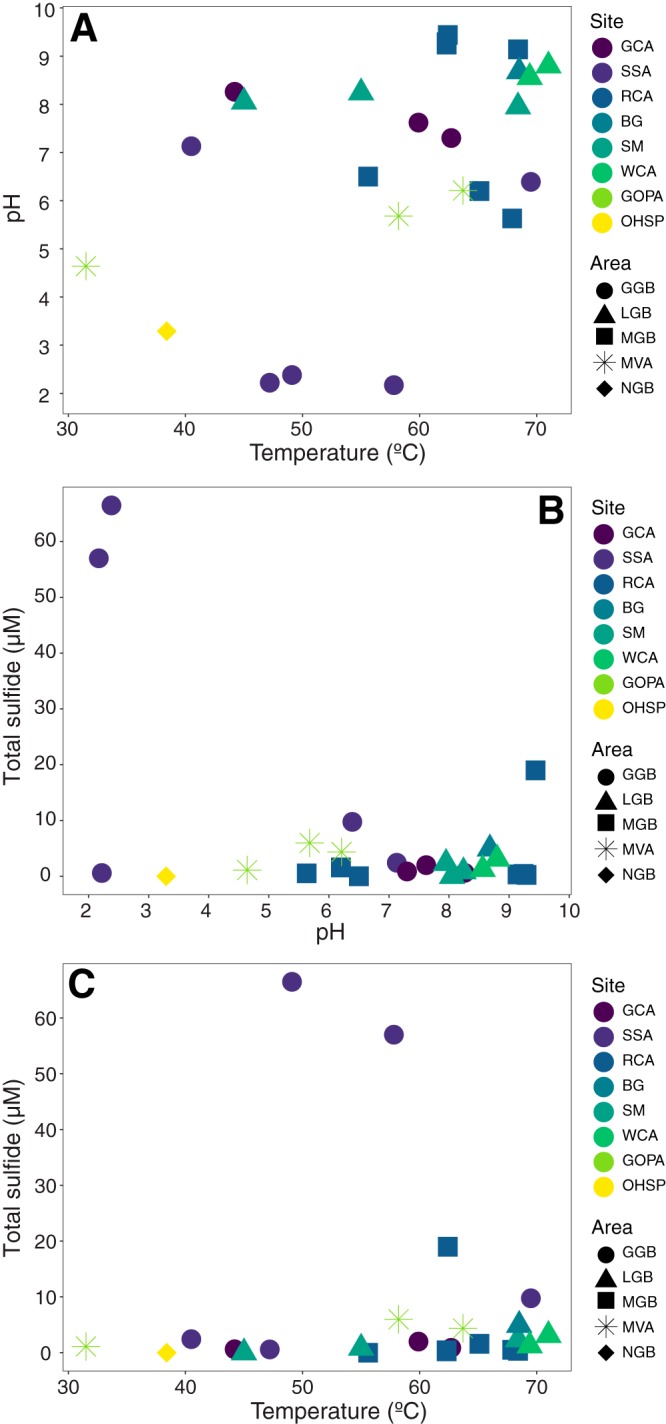
Sites targeted for amplicon sequencing plotted as a function of temperature and pH (A), sulfide and pH (B), and sulfide and temperature (C). Site and area designations are provided in Table S1.

We collected filaments and microbial mats/biofilms (*n* = 27; see examples in Fig. S1 in the supplemental material) from sites that span a pH range of 2.2 to 9.4 and a temperature range from 31.5°C to 71.0°C (Fig. 1A and B and Table S1). Considering reduced species that could support anoxygenic photosynthesis, i.e., light-driven carbon fixation in the absence of oxygen generation, sulfide concentrations in the sites ranged from below the detection limit (∼150 nM) to ∼66 μM, whereas ferrous iron concentrations ranged from below the detection limit (∼180 nM) to ∼64 μM. Most sites had sulfide concentrations below 10 μM and Fe^2+^ concentrations below 5 μM (Fig. 1C and Table S1). Total arsenic concentrations ranged from 75 nM to 21 μM, but we cannot discern the fraction of arsenic in the reduced state from the inductively coupled plasma mass spectrometry data. Dissolved inorganic carbon (DIC) concentrations ranged from 31 μmol/liter to 488 μmol/liter (Table S1). We did not measure NO_2_^−^ or H_2_. Anoxygenic phototrophs using NO_2_^−^ as an electron donor have not yet been reported in hot springs, whereas hydrogen has been implicated as a substrate for photoautotrophic growth in *Chloroflexi* in alkaline mats (54).

10.1128/mSystems.00498-19.1 FIG S1 Examples of filaments (A to F) and microbial mats/biofilms (G to L) sampled in this study. Download FIG S1, PDF file, 2.2 MB. Copyright © 2019 Hamilton et al. 2019 Hamilton et al. This content is distributed under the terms of the Creative Commons Attribution 4.0 International license.

10.1128/mSystems.00498-19.8 TABLE S1 GPS coordinates, pH, conductivity, temperature, aqueous geochemistry, and stable isotope analyses. Total carbon and nitrogen and stable isotope analysis results for biomass and dissolved inorganic carbon (DIC), DIC δ^13^C values, dissolved organic carbon (DOC), and DOC δ^13^C values are also provided. Na, K, Ca, and Mg were determined via inductively coupled plasma optical emission spectroscopy. Sample type is indicated: fil, filament or mat, microbial mat/biofilm. Des., sample designator; Sulf, sulfide; nd, not determined; bdl, below detection limit. Detection limits: sulfide, 5 μg/liter S^2−^; Fe^2+^, 20 μg/liter. Library names for the 16S rRNA amplicon libraries included in the present study are provided. All sequence data, including raw reads with quality scores and mapping data for this study, are available from the NCBI Sequence Read Archive (SRA) database under with the BioProject number PRJNA513338. Download Table S1, XLSX file, 0.02 MB. Copyright © 2019 Hamilton et al. 2019 Hamilton et al. This content is distributed under the terms of the Creative Commons Attribution 4.0 International license.

**FIG 2:**
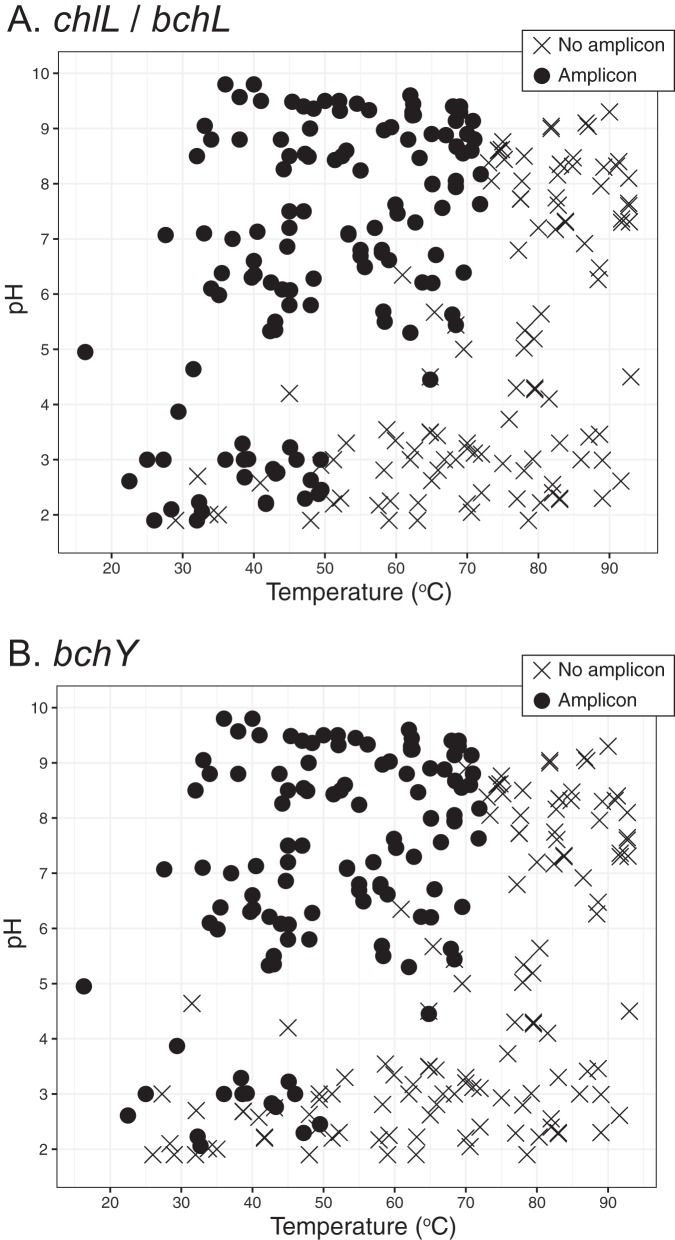
Occurrence of *chlL* and *bchL* (A) and *bchY* (B) across pH and temperature. The presence (filled circles) or absence (X) of *chlL* and *bchL* (A) and *bchY* (B) in 289 samples from YNP is plotted as a function of pH and temperature. One hundred five samples are from reference 5. The rest are from this study or unpublished data. *chlL* and *bchL* are proxies for oxygenic and anoxygenic phototrophs, whereas *bchY* is encoded by some anoxygenic phototrophs. Site and area designations are provided in Table S1.

**(i) Distribution of *bchY*, a proxy for anoxygenic phototrophs.** Anoxygenic phototrophs occur throughout temperature and pH ranges similar to those of oxygenic phototrophs, but anoxygenic phototrophs are less prevalent at low pH. In this study and both previous and unpublished work (5), we have screened DNA extracts from 289 sediment, filament, floc, and mat samples collected from hot springs in YNP that range in pH from 1.9 to 9.8 and temperature from 16.3°C to 93.0°C for the presence of *chlL/bchL* and *bchY*. *chlL/bchL* encode a protein necessary for pigment biosynthesis in both oxygenic and anoxygenic phototrophs, while *bchY* is a gene encoding a protein involved in the biosynthesis of BChls *a*, *b*, and *g* in anoxygenic phototrophs. Both *chlL/bchL* and *bchY* were detected in nearly all samples above pH 6 at a temperature lower than 72°C (Fig. 2). Below pH 5, *bchY* was recovered from a subset of samples where *chlL* and *bchL* were also detected. As expected, *bchY* was only detected in samples where *chlL* and *bchL* were also detected (*chlL/bchL* genes are found in both oxygenic and anoxygenic chlorophototrophs, but *bchY* is only found in anoxygenic phototrophs [41]). This distribution pattern is consistent with our previous observations and those of others (3), where anoxygenic phototrophs are more constrained by temperature and pH than oxygenic phototrophs.

**FIG 3:**
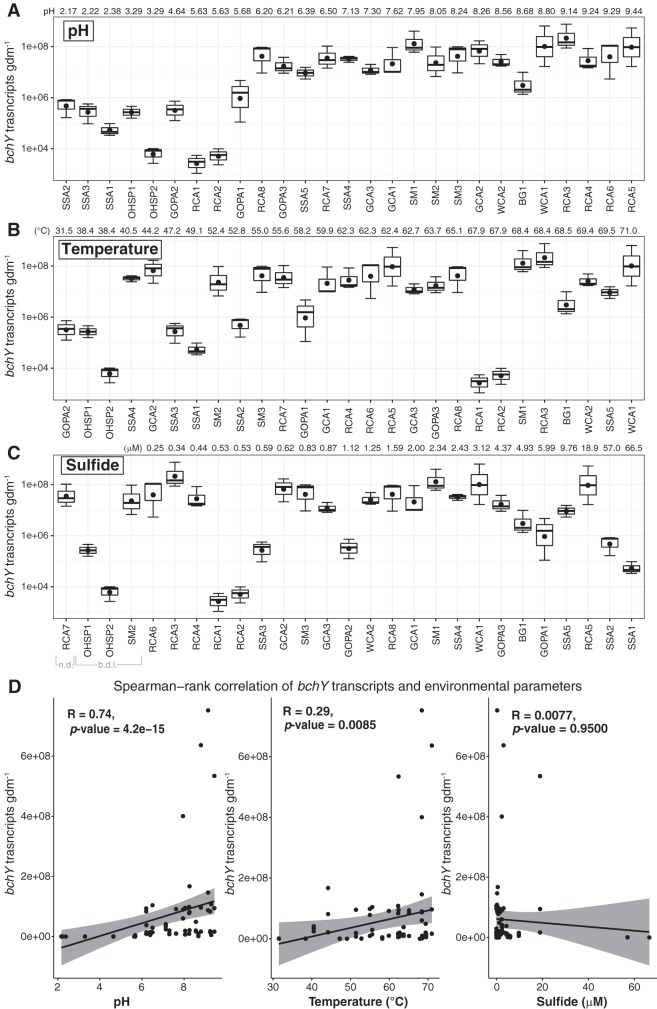
*bchY* transcript abundance across the range of pH (A), temperature (B), and sulfide concentration (C) in our sample sites. (D) Spearman rank correlation tests between *bchY* transcript abundance and environmental parameters (pH, temperature, and sulfide). Physicochemical data and sample designations are provided in Table S1. Boxplots displaying the median and quartiles from triplicate measurements. n.d., not detected; b.d.l., below detection limit (5 μg/liter S^2−^).

**(ii) Abundance of *bchY* transcripts by qRT-PCR.** We observed a range in *bchY* transcript abundance from 3.2 × 10^3^ to 1.5 × 10^8^ copies per gram of dry mass (gdm) (Fig. 3). In general, *bchY* transcripts were more abundant at higher pH (pH >6) (Fig. 3A). Most samples above pH 6 contained at least 10^6^ transcripts of *bchY*, while all samples below pH 5.6 contained fewer than 10^6^ *bchY* transcripts. In samples with pH >6, *bchY* transcript abundance ranged from 1.5 × 10^6^ to 1.5 × 10^8^ from 40.5°C to 71.0°C. *bchY* transcripts were lowest in sites below 50°C (<10^6^), except for Sylvan Springs Area 4 (SSA4) and Geyser Creek Area 2 (GCA2) (Fig. 3B). At 71.0°C, we recovered 10^7^ *bchY* transcripts, suggesting anoxygenic phototrophs are active near the upper temperature limit of photosynthesis (∼72 to 73°C) (Fig. 2). Based on Spearman rank‐order correlation, there was a significant positive relationship between increasing *bchY* transcript abundance and pH as well as temperature (*P* > 0.05) (Fig. 3D). In contrast, no pattern was observed between *bchY* transcript abundance and sulfide concentration; transcript abundance values of 10^8^ were observed in the samples with no detectable sulfide (below detection limit) and in samples with up to 19 μM sulfide (Fig. 3C). The lowest abundances of *bchY* transcripts were observed in Rabbit Creek Area 1 (RCA1) and RCA2 (pH 5.63, 58.2°C, and 0.53 μM sulfide) and One Hundred Springs Plain 2 (OHSP2) (pH 6.39, 69.5°C, 9.76 μM sulfide). Despite the low abundance of *bchY* transcripts, these conditions (in terms of pH and temperature) are suitable for photosynthesis based on the distribution of *chlL/bchL* and *bchY* (Fig. 2) and the recovery of *bchY* transcripts from sites with similar geochemistry (Fig. 2). An increased abundance of *bchY* transcripts at alkaline pH is consistent with previous DNA-based studies where copies of the *bchY* gene were highest in alkaline hot springs (5). In that study, the abundance of *bchY* genes was not significantly correlated with environmental geochemistry. High abundance of *bchY* transcripts in ocean surface waters suggests anoxygenic phototrophs in this environment are of ecological importance (55). In circumneutral hot springs, transcripts of Bchl biosynthesis genes have been reported, but peak transcript abundance varies by site, taxonomy, or time of day (18, 56, 57). Given high abundance of *bchY* transcripts in alkaline sites reported here, our data suggest anoxygenic phototrophs are ecologically relevant, particularly in alkaline hot springs.

**FIG 4:**
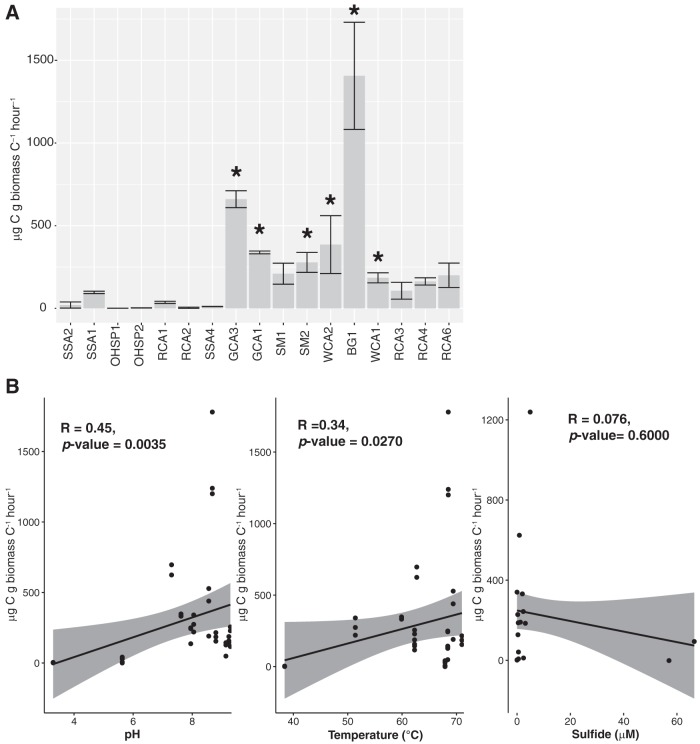
Rates of anoxygenic photosynthesis arranged in order of increasing pH. Error bars from triplicate measurements. Asterisks indicate rates of carbon uptake in the presence of DCMU that are statistically different (*P < *0.05) from the light and dark treatments (rates and *P* values are provided in Table S2).

**(iii) Anoxygenic photosynthesis activity.** We employed *in situ* microcosms (in the presence of NaH^13^CO_3_) to assess assimilation of inorganic carbon (autotrophy) in a subset of samples (*n* = 17) across the range of pH and temperature of our sample sites. Light-dependent inorganic carbon assimilation (photoautotrophy and photosynthesis) was significantly higher than that of dark assimilation (chemoautotrophy) in all sites except Sentinel Meadows 1 (SM1) (Table S2), suggesting the sites we targeted support active photosynthesis. To assess the potential for assimilation of inorganic carbon via anoxygenic photosynthesis, we performed assays in the light in the presence of 3-(3,4-dichlorphenyl)-1,1-dimethylurea (DCMU), a photosystem II (PSII) inhibitor. In all assays performed in the light, rates of assimilation of inorganic carbon were significantly higher in unamended assays than in assays amended with DCMU (Fig. 4A and Table S2). Assuming assays performed in the light account for all primary productivity, in the circumneutral to alkaline sites, rates of photoassimilation in the presence of DCMU ranged from 3.5% to ∼31% of the total primary productivity (difference between light and DCMU conditions). In assays amended with DCMU, where rates of photoassimilation were significantly higher than those performed in the dark, rates of anoxygenic photosynthesis ranged from 11 μg C uptake/g C biomass/h in site OHSP1 to 1,406 μg C uptake/g C biomass/h in site Boulder Geyser 1 (BG1) (Fig. 4A and Table S2). We observed DCMU-dependent photoassimilation at low pH (SSA1, pH 2.38, temperature of 49.1°C) and high temperature (White Creek Area 1 [WCA1], pH 8.80, temperature of 71.0°C) (Fig. 4 and Table S1), indicating anoxygenic photosynthesis is active across a wide range of pH and temperatures. However, in SSA2, OHSP1, OHSP2, RCA1, RCA4, and RCA6, rates of assimilation in the presence of DCMU were indistinguishable from dark assimilation, which indicates PSII-independent photosynthesis is not active in these sites (Table S2). These sites varied in temperature and pH (and sulfide), suggesting other environmental parameters or a combination of factors inhibit the activity of anoxygenic photoautotrophy. Based on Spearman rank‐order correlation, there was a significant positive relationship between increasing rates of anoxygenic photoautotrophy and pH as well as temperature (Fig. 4B). Similar to the abundance of *bchY* transcripts, there was no relationship between carbon assimilation in the presence of DCMU and sulfide concentration.

10.1128/mSystems.00498-19.9 TABLE S2 Rates of carbon assimilation and *P* values for *t* tests for each comparison of C uptake rates. *P* values in boldface are below 0.05. All carbon isotope values are given as absolute values. All samples have ^13^C-labeled bicarbonate added. Light, labeled bicarbonate added; DCMU, DCMU added; Dark, aluminum foil wrapped. St. dev., standard deviation of assays performed in triplicate. Treat., amendment. Site and area designations are provided in Table S1. Download Table S2, XLSX file, 0.01 MB. Copyright © 2019 Hamilton et al. 2019 Hamilton et al. This content is distributed under the terms of the Creative Commons Attribution 4.0 International license.

**(iv) Diversity of anoxygenic phototrophs.** Alpha diversity patterns for the overall microbial community and the putative phototroph fraction of the community are similar: the acidic sites had slightly higher alpha diversity values than alkaline sites independent of the temperature when either the full data set (Fig. S3A) or only operational taxonomic units (OTUs) affiliated with phototrophic taxa were considered (Fig. S3B). Mildly acidic to neutral pH hot springs (pH ∼4 to 6) resulting from reduced vapor phase-influenced water enriched in volcanic gases with oxidized near-surface meteoric water have been proposed to host highly diverse chemosynthetic communities (58). A subset of our samples fell within the range of pH ∼4 to 6, and we recovered abundant (ranging from 5 to 25%) OTUs assigned only at the phylum level as “Bacteria_unclassified” or “Other” (Fig. S4). These data indicate high diversity can persist in mildly acidic to neutral pH site systems where anoxygenic phototrophs are present and active (e.g., Fig. 2 to 4). Here, the highest rates of photoassimilation were observed in sites with low pH and low alpha diversity—specifically SSA1 and SSA2 (6,072 and 6,736 μg C uptake/g C biomass/h, respectively) (Table S2). On large spatial scales, increasing photosynthetic carbon fixation has been correlated to increasing biodiversity (59); however, in marine systems decreasing diversity has been observed in highly productive sites (60, 61). This relationship does not hold at small spatial scales. In terrestrial geothermal springs, diversity typically decreases with pH extremes including low pH (62), presumably due to the physiological adaptations necessary to thrive in acidic environments. A combination of elevated pH, temperature, or other geochemical variables could limit diversity in increasing alkaline hot springs.

10.1128/mSystems.00498-19.3 FIG S3 Alpha diversity statistics for the 27 amplicon libraries arranged by increasing pH. (A) Alpha diversity metrics for the complete data set. (B) Alpha diversity statistics for sequences affiliated with characterized phototrophs. Physicochemical data and sample designations provided in Table S1. Download FIG S3, PDF file, 0.2 MB. Copyright © 2019 Hamilton et al. 2019 Hamilton et al. This content is distributed under the terms of the Creative Commons Attribution 4.0 International license.

10.1128/mSystems.00498-19.4 FIG S4 16S rRNA gene sequences binned at the phylum (A), class (B), and genus (C) levels recovered from samples across a range of geochemical spaces in YNP. Site and area designations are provided in Table S1. Classification was based on Silva (v132) reference taxonomy as described in Materials and Methods. Download FIG S4, PDF file, 0.3 MB. Copyright © 2019 Hamilton et al. 2019 Hamilton et al. This content is distributed under the terms of the Creative Commons Attribution 4.0 International license.

### Putative anoxygenic phototrophs and their potential metabolism.

**FIG 5:**
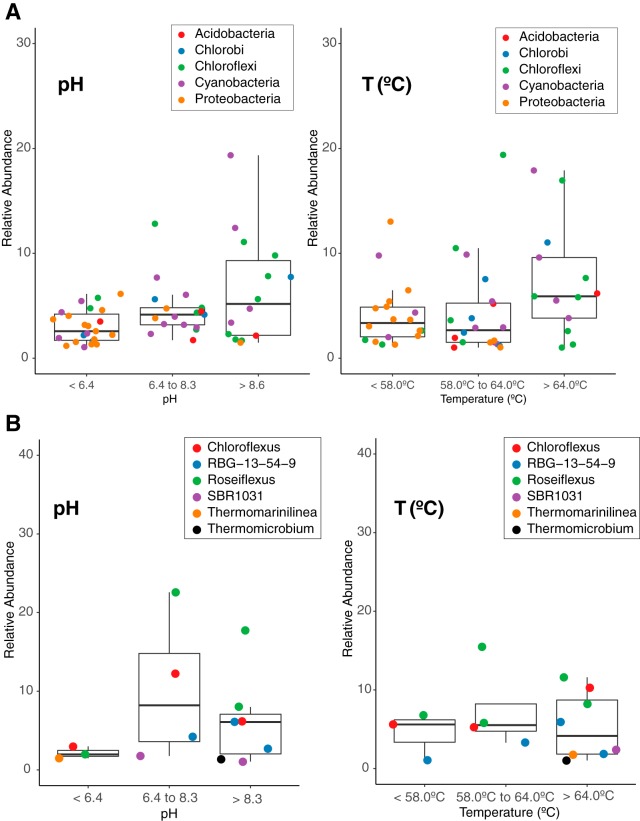
Relative abundance of OTUs affiliated with characterized phototrophs (A) and relative abundance of OTUs affiliated with *Chloroflexi* (B) in the 27 sites in YNP. Sites were grouped into three pH groups (pH <6.4, *n* = 12 sites; pH 6.4 to 8.3, *n* = 8 sites; and pH >8.3, *n* = 7 sites) and three temperature groups (<58°C, *n* = 11 sites; 58°C to 64°C, *n* = 7 sites; and >64°C, *n* = 9 sites). Relative abundances of OTUs in the three pH and temperature groups are plotted over boxplots displaying the median and quartiles of each group (OTUs with relative abundance of <1 were omitted to avoid overplotting). Points are shaded by phylum or class in panel A or the highest-level classification available for *Chloroflexi* in panel B.

We recovered sequences affiliated with all seven bacterial phyla with characterized phototrophic members (Fig. 5A and Fig. S4A); however, sequences affiliated with the potential phototrophs within the *Gemmatimonadetes* and *Firmicutes* were rare. OTUs affiliated with characterized phototrophs within the *Proteobacteria* were more prevalent (but not abundant) at low pH (<6.4) and lower temperature (<58°C) (Fig. 5A and Fig. S4). Sequences affiliated with *Chlorobi* (in the phylum *Bacteriodetes*) were more abundant in sites with pH >6.4 and temperature of >58°C (Fig. 5A and Fig. S4). We observed abundant OTUs affiliated with characterized phototrophic *Chloroflexia*, including *Chloroflexus* and *Roseiflexus*, particularly in circumneutral and alkaline sites (Fig. 5B and Fig. S4). Sequences affiliated with *Roseiflexus* were abundant in GCA1, all RCA sites except RCA2, all SM sites, and WCA1, whereas sequences affiliated with *Chloroflexus* were observed in BG1, GCA1, RCA1, RCA8, and all the SM sites (Fig. S4). We only recovered a small number of OTUs affiliated with phototrophic *Acidobacteria*. These OTUs were observed in sites with pH >6.4 and across the range of temperatures (Fig. 4A). Sequences affiliated with phototrophic *Cyanobacteria* (*Oxyphotobacteria*) were recovered from sites across the range of temperatures and pH (Fig. 5A and Fig. S4). There are only a few examples of *Oxyphotobacteria* that can perform anoxygenic photosynthesis, and the trait does not appear to be constrained across closely related taxa. As a result, we cannot predict facultative anoxygenic photosynthesis activity from our *Oxyphotobacteria* 16S rRNA gene sequences.

OTUs affiliated with *Chloroflexus* and *Roseiflexus* were recovered from circumneutral to alkaline sites (Fig. 5B). Characterized *Roseiflexus* spp. grow phototrophically under oxic conditions, whereas species of the genus *Chloroflexus* may grow phototrophically under oxic (C. aurantiacus) or anoxic (C. aggregans) conditions (63–65). There are reports of phototrophic *Chloroflexi* outside the *Chloroflexia*, including the recently proposed new class-level clade “*Candidatus* Thermofonsia” (66), which includes “*Candidatus* Roseilinea gracile,” described from a metagenome from a YNP hot spring (6, 67). We recovered sequences affiliated with “*Ca*. Thermofonsia” (SBR1031 in Fig. 5B), but these OTUs were not abundant. Sequences affiliated with Chloracidobacterium thermophilum, a member of the *Acidobacteria*, were observed in most of the circumneutral to alkaline sites (Fig. S4). *C. thermophilum* is an aerobic photoheterotroph (68) that has been observed in alkaline springs ranging in temperature from ∼50 to 65°C in YNP (25, 57, 69). Sequences affiliated with photoautotrophic *Chlorobi* were also abundant in circumneutral to alkaline sites, which is consistent with previous studies, including metagenomic data from Octopus and Mushroom Springs in YNP (57, 67). Most described *Chlorobi* are anaerobic obligate photoautotrophs. Sequences affiliated with a recently described aerobic heterotrophic *Chlorobi*, “*Ca.* Thermochlorobacteriaceae bacterium” GBChlB (70), were recovered from the alkaline RCA samples and SM1 and SM2 (Fig. S4), and an OTU affiliated with GBChlB was among the top 50 OTUs recovered (Fig. 5). “*Ca.* Thermochlorobacteriaceae bacterium” GBChlB is closely related to “*Ca.* Thermochlorobacter aerophilum,” which was initially described in alkaline terrestrial hot springs in YNP (57). Both are aerobic phototrophic members of *Chlorobi* that do not appear to oxidize sulfur. We also observed small numbers of 16S rRNA gene sequences assigned to the proposed phylum “*Candidatu*s Palusbacterota” (formerly phylum WPS-2) (Fig. S4) (71). In metagenomic data, members of “*Candidatu*s Palusbacterota” encode type II reaction centers, and members of this proposed phylum are thought to inhabit acidic, aerobic environments.

*Ectothiorhodospira*, a genus of anoxygenic purple phototrophs within the *Gammaproteobacteria*, were present in SSA1, SSA2, and SSA3 (Fig. S4 and S5). *Ectothiorhodospira* spp. are typically observed in alkaline systems (72), whereas these SSA sites are all acidic (pH <2.4) (Table S1). We also recovered sequences affiliated with *Rhodopila* (within the *Alphaproteobacteria*) from SSA1 and SSA3 (Fig. S4 and S5). A member of this genus, Rhodopila globiformis, was isolated from an acidic hot spring in YNP (pH ∼3) and is the most acidophilic anaerobic anoxygenic phototrophic purple bacterium characterized to date (73). Sequences affiliated with the *Acidiphilium*, a genus of aerobic anoxygenic phototroph within the *Alphaproteobacteria* (74), were abundant in sites with pH <6.4 (Fig. S5). In OHSP2, *Acidiphilium* OTUs accounted for ∼30% of the total sequences (Fig. S4).

10.1128/mSystems.00498-19.5 FIG S5 Relative abundance of OTUs affiliated with *Proteobacteria*. Relative abundance of OTUs in the three pH (A) and temperature (B) groups are plotted over boxplots displaying the median and quartiles of each group (OTUs with relative abundance of <1% were omitted to avoid overplotting). Download FIG S5, PDF file, 0.1 MB. Copyright © 2019 Hamilton et al. 2019 Hamilton et al. This content is distributed under the terms of the Creative Commons Attribution 4.0 International license.

Despite the physiological diversity of phototrophic *Proteobacteria*, sequences affiliated with known phototrophic *Proteobacteria* were rare in sites above pH 6.4 and sites with temperatures of >58.0°C (Fig. S5). Phototrophs within the phylum *Proteobacteria* have been observed in a number of alkaline systems, including both oxic and anoxic systems. For instance, aerobic heterotrophic phototrophs have been described from mats with pH ranging from 8.0 to 9.4 (74). Aerobic anoxygenic phototrophic strains from the hydrothermal vents at Juan de Fuca Ridge grew under a large range of temperatures (5 to 42°C) and pH (5.5 to 10). Proteobacterial phototrophs have also been observed in brackish stratified lakes with environments with exceptionally high sulfide, including Mahoney Lake, British Columbia, and Fayetteville Green Lake, New York (75, 76). Isolates of phototrophic *Proteobacteria* also have been described from brackish terrestrial hot springs in Japan at pH 5.8 and 42°C (74), and aerobic anoxygenic phototrophs are widespread in the open ocean (77) and saline lakes (15).

**FIG 6:**
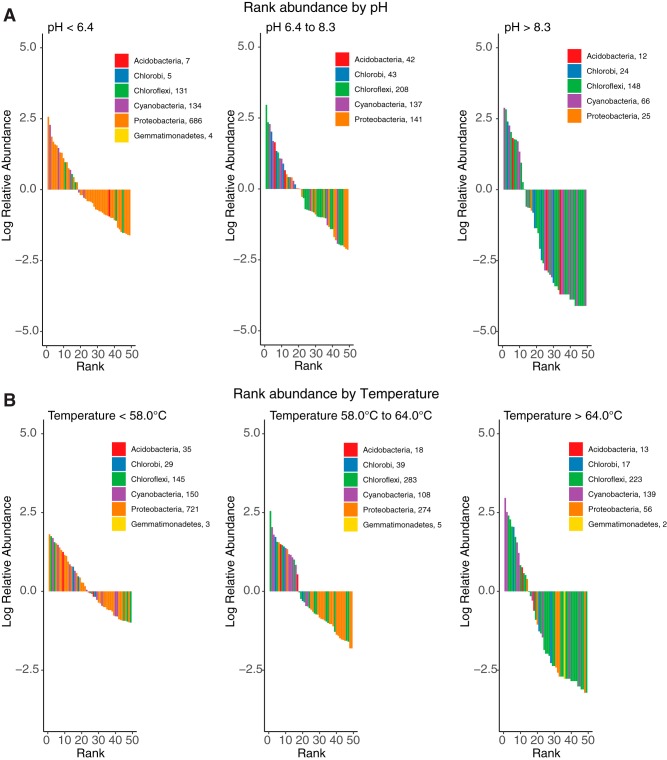
Rank abundance plots of the top 50 OTUs in sites across pH (A) and temperature (B) space in our samples. Sites were grouped into three pH groups (pH <6.4, *n* = 12 sites; pH 6.4 to 8.3, *n* = 8 sites; and pH >8.3, *n* = 7 sites) and three temperature groups (<58°C, *n* = 11 sites; 58°C to 64°C, *n* = 7 sites; and >64°C, *n* = 9 sites). OTUs were ranked by decreasing relative abundance in each pH or temperature group; the top 50 OTUs are shown with bars shaded by phylum (or class in the case of *Chlorobi*).

Rank abundance plots of the top 50 most abundant OTUs from phototrophic taxa reflects the patterns observed in the overall community composition data (Fig. 6). For instance, OTUs affiliated with *Proteobacteria* were prevalent at low pH and below 58°C, whereas *Chloroflexi*, *Chlorobi*, and *Cyanobacteria* OTUs were more abundant at pH >6.4 and above 64°C. While we recovered several *Chloroflexi* OTUs in sites above pH 8.3, the majority of OTUs affiliated with *Chloroflexi* were recovered from sites with pH <8.3. Above 64°C, more than half of the top 50 OTUs were assigned to *Chloroflexi*. These observations suggest circumneutral springs with temperatures of >64°C favor multiple populations of *Chloroflexi*.

### Cooccurrence of oxygenic and anoxygenic phototrophs.

Our data indicate oxygenic and anoxygenic phototrophs coexist in mats, filaments, and sediments across temperature and pH gradients in YNP. We observed DCMU-dependent inorganic carbon assimilation (anoxygenic photosynthesis) only in sites where light-dependent photoassimilation was also observed. This observation is consistent with other studies of circumneutral geothermal systems, including high-spatial-resolution characterization of mats in the Lower Geyser Basin (6), but has not been reported for low-pH sites. The potential anoxygenic phototrophs that were prevalent at low pH and low temperature were most closely related to aerobic anoxygenic phototrophs within the *Proteobacteria* (Fig. S4). These sequences were rare or not observed in sites above pH 6.4 despite the prevalence of anoxygenic phototrophs within the *Proteobacteria* in circumneutral temperate systems, including aquatic habitats and ocean waters (73). Sequences affiliated with aerobic anoxygenic phototrophs cooccur with algae in acid mine drainage sites with pH as low as 2.1 (78). These studies and our data suggest low-pH systems select for cooccurring algae and aerobic anoxygenic phototrophs or facilitate coexistence of these populations, but the nature of these interactions requires further study.

In higher temperature circumneutral and alkaline hot springs, *Chloroflexi* and *Oxyphotobacteria* were the most abundant putative phototrophs. Cooccurrence of *Chloroflexi* and *Cyanobacteria* has been reported in microbial mats between a 50°C to 65°C temperature range in terrestrial hot springs in Thailand (79). Previous studies have reported a cross-feeding relationship between *Chloroflexi* and *Cyanobacteria* (80), where *Cyanobacteria* fix carbon for heterotrophic *Chloroflexi*. Furthermore, a metatranscriptomics study of mat communities in Mushroom Spring in YNP suggests *Chloroflexi* perform phototmixotrophy during the day, store organic carbon from *Cyanobacteria* as glycogen, and deplete glycogen stores via fermentation at night (18). Our data are consistent with a relationship between *Chloroflexi* and *Cyanobacteria* across temperature gradients where phototrophic members of the two phyla are abundant.

### Carbon isotope fractionation and inorganic carbon assimilation rates.

**FIG 7:**
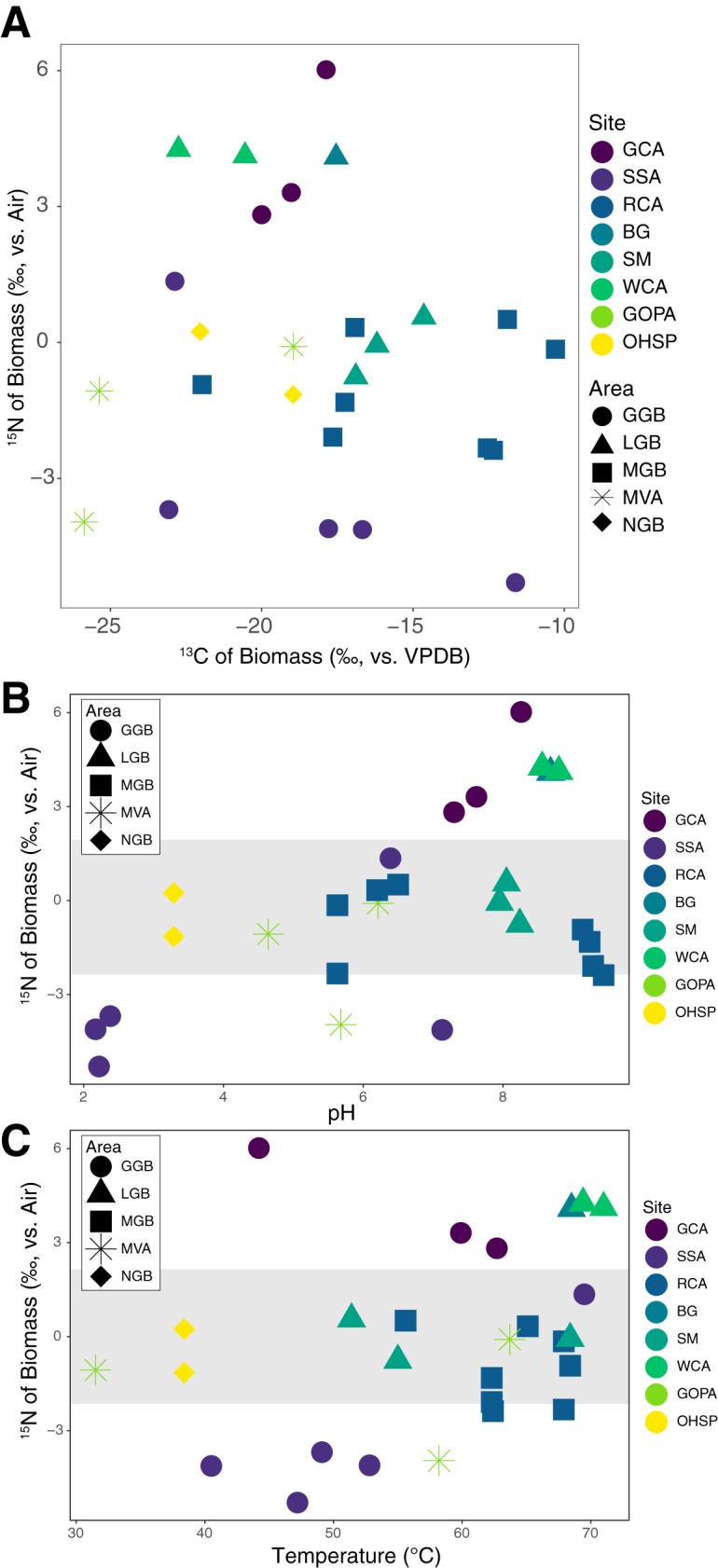
Carbon (A) and nitrogen (B and C) stable isotope results for biomass samples. Nitrogen-stable isotope results for biomass samples are plotted against pH (B) and temperature (C). Site and area designations are provided in Table S1. The gray bar in panels B and C indicates values typically observed for biological nitrogen fixation.

Microbes fractionate stable isotopes during metabolism and growth, resulting in characteristic isotope signatures in biomass. For instance, the known carbon fixation pathways can exhibit different carbon isotope fractionation values, and nitrogen transformations, including biological nitrogen fixation, impart isotopic signals reflected in the resulting generated biomass (51). In our filament and mat biofilm samples, δ^13^C values ranged between −25.87 and −10.29‰ and δ^15^N values ranged between −4.13 and 6.02‰ (Fig. 7 and Table S1).

We did not observe any general trends between biomass δ^13^C values and geochemistry or morphology (filaments or mat biofilm), which is consistent with other hot spring studies (e.g., see references 26 and 51). For instance, biomass samples from RCA ranged in δ^13^C values between −21.97 and −10.29‰ and SSA samples ranged from −23.07 to −11.61‰. Biomass from alkaline sites at Geyser Creek Area (GCA) and White Creek Area (WCA) had δ^13^C values that ranged from −20.00 to −17.87‰ and −22.75 to −20.55‰, respectively. The three samples from SM had δ^13^C values of −19.07, −16.19, and −14.64‰. Two samples from the Mud Volcano Area (MVA), Greater Obsidian Pool Area 1 (GOPA1) and GOPA2, had the lightest biomass δ^13^C values, at −25.37 and −25.87‰, respectively. These large ranges in biomass δ^13^C values could be attributed to fractionation from DIC sources varying in δ^13^C values, with measured δ^13^C values of DIC ranging from −3.74 to 4.30‰. Thus, calculating fractionation factors is valuable for removing this effect.

In general, δ^15^N values close to the value of atmospheric air (0‰ ± 2‰) indicate most of the N incorporated in the biomass is sourced from nitrogen fixation, while more positive values are associated with limited fixed N from allochthonous sources and/or volatilization of NH_3_ and more negative values with subsurface boiling concentrating isotopically light NH_3_ (e.g., see references 51, 81, and 82) (Fig. 7B and C). Low-pH sites tended to have biomass with more negative δ^15^N values while circumneutral to alkaline sites tended to have biomass with more positive δ^15^N values, and values close to 0‰ were found across the pH value range (Fig. 7B and C and Table S1).

**FIG 8:**
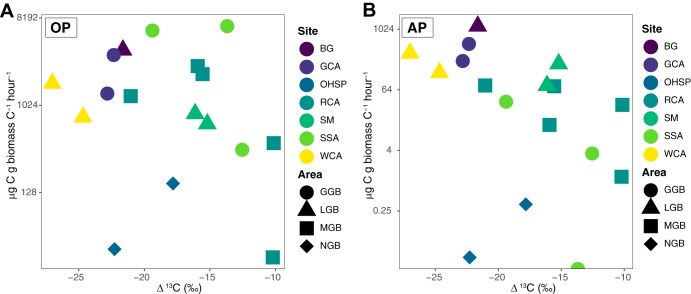
Carbon isotopic fractionations in biomass plotted against rates of oxygenic (A) and anoxygenic photosynthesis (B), OP and AP, respectively. Site and area designations are provided in Table S1.

We examined carbon isotope fractionation (Δ^13^C) by calculating the difference between the biofilm *δ*^13^C and DIC *δ*^13^C. Δ^13^C values ranged from −27.01 to −9.96‰ (Fig. S6). In sites where both oxygenic and anoxygenic photosynthesis were observed, Δ^13^C ranged from −10 to −27‰, and there was no clear pattern of Δ^13^C values with increasing rates of photosynthesis (Fig. 8) or with temperature, pH, or sulfide (Fig. S6). In general, circumneutral and alkaline sites exhibited higher rates of oxygenic and anoxygenic photosynthesis in higher temperature sites and were characterized by larger Δ^13^C values (BG, WCA, and GCA) (Fig. 8). Abiotic factors can also impact Δ^13^C: the availability of CO_2_ can affect carbon fractionation as well as changes in flow and aerial exposure. The preferred substrate for bacterial photosynthesis, bicarbonate (83, 84), in these systems is a function of the CO_2_ availability, which is in turn a function of subsurface processes (85). At pH 7 to 8, the majority of dissolved inorganic carbon is in the form of HCO_3_^−^, and DIC concentration typically decreases and pH increases down outflow channels with distance from the source due to CO_2_ outgassing (i.e., HCO_3_^−^→CO_2(g)_ + OH^−^).

10.1128/mSystems.00498-19.6 FIG S6 Carbon isotopic fractionations in biomass mass collected for the present study. Values are plotted against pH (A) and temperature (B) and with increasing pH (C), increasing temperature (D), and increasing sulfide concentration (E). n.d., not detected; b.d.l., below detection limit (5 μg/L S^2−^). Download FIG S6, PDF file, 0.2 MB. Copyright © 2019 Hamilton et al. 2019 Hamilton et al. This content is distributed under the terms of the Creative Commons Attribution 4.0 International license.

Fractionation also could be dependent upon phototrophic biofilm/mat thickness and/or coherency and flow regime. With increasing mat thickness from a robust oxygenic photosynthetic community (or increasing coherency/decreasing flow intensity), it is harder for DIC to diffuse into the mat. Thus, the release of CO_2_ through heterotrophic breakdown (e.g., heterotrophy, fermentation, and photoheterotrophy) of organic carbon in the deeper mat layers could become an increasingly important source of DIC for anoxygenic phototrophs. Thus, a thick mat generated by oxygenic phototrophs would create increasing niche space for anaerobic heterotrophs and anoxygenic photoautotrophs, and if this is the case, then we would expect to see a cooccurrence of oxygenic phototrophs and anoxygenic phototrophs. A systematic study of these biotic and abiotic factors is necessary to determine the cause for variation in the signal and the positive relationship observed between rates of anoxygenic photosynthesis and increasing Δ^13^C. Regardless, δ^13^C and Δ^13^C signals reported in this study are consistent with those preserved in the rock record (e.g., see reference 86). Our data underscore a potential role for diverse phototroph physiology in contributing to these signals across space and time and a need for systematic characterization of the contribution of anoxygenic phototrophy to microbial community biomass δ^13^C and Δ^13^C signals.

### Potential for photoheterotrophic activity at low pH.

We did not observe anoxygenic photosynthesis in sites below pH 6. However, we did detect *bchY* genes and recover *bchY* transcripts and 16S rRNA gene sequences affiliated with phototrophic *Cyanobacteria* and anoxygenic phototrophs from sites below pH 6 (Fig. 2 to 4), suggesting these populations are present and active under acidic conditions. *Cyanobacteria* were not abundant at low pH and likely were not responsible for the elevated rates of light-dependent photoassimilation observed in the acidic SSA sites (Table S2). We also did not quantify photoheterotrophy (or photomixotrophy) but recovered abundant OTUs related to photoheterotrophic (or photomixotrophic) members of the *Proteobacteria*, *Chlorobi*, *Acidobacteria*, and *Chloroflexi*. At low pH, the majority of the inorganic carbon is in the form of aqueous CO_2_, a substrate for acidophilic photoautotrophic algae, including eukaryotic red algae of the *Cyanidioschyzon*, *Cyanidium*, and *Galdieria*, which are abundant in these environments in YNP (7–9). Low pH may constrain the distribution of bacterial photoautotrophs, which prefer bicarbonate as a substrate for inorganic carbon photoassimilation. In environments with acidic pH, phototrophs are restricted to temperatures of less than ∼56°C (Fig. 2) (3–5, 7, 9, 40, 87). This is presumably due to sulfide-dependent suppression of phototrophic activity (specifically, photosystem II) in algae, the predominant phototrophs in springs with pH <5.0 (8, 40, 88). Notably, the SSA sites with elevated rates of photoassimilation (Table S2) had sulfide concentrations of >57 μM, which suggests the inhibitory effect of sulfide on algae requires higher sulfide concentrations than previously reported (i.e., 5 μM [3]). Regardless, our amplicon data underscore a role for photoheterotrophy (and/or photomixototrophy), particularly in low-pH hot springs. The recovery of multiple populations most closely related to aerobic photoheterotrophic taxa is consistent with photoassimilation of inorganic carbon via oxygen-producing photosynthesis through the activity of *Oxyphotobacteria* at circumneutral to alkaline pH and algae in low-pH systems. The production of oxygen via oxygenic photosynthesis could be of particular importance for aerobic phototrophic taxa at high temperature, where O_2_ solubility is low. Still, we did observe significant inorganic carbon uptake via anoxygenic photosynthesis in several sites where H_2_S is a possible electron donor, including sites with temperatures above 68°C (Fig. 3).

### A combination of biotic and abiotic factors constrains the distribution of anoxygenic phototrophs.

**FIG 9:**
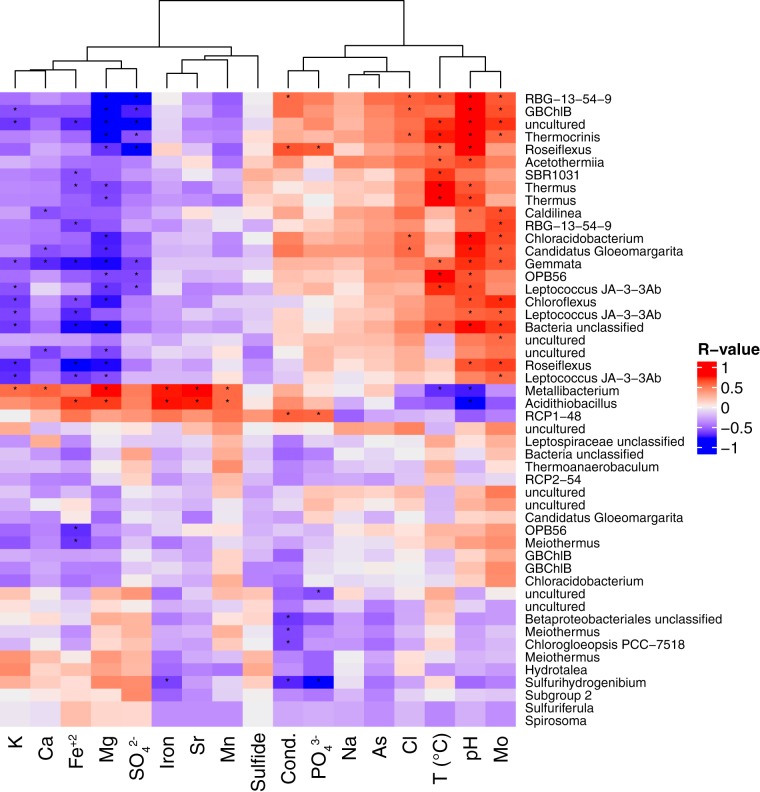
Heatmap of Spearman correlation coefficient (Spearman’s rho) values between rarefied abundances of the top 50 most abundance OTUs classified at the genus level and by environmental variables. *P* values of <0.01 are indicated with an asterisk.

Temperature and pH were important determinants of richness in our samples; we observed an inverse relationship between diversity and temperature in concordance with previous work (38). When considering only the top 50 OTUs, which accounted for >60% of the total sequences recovered, significant positive correlations were observed between pH and temperature and the top 50 OTUs, whereas sulfate, Mg, Fe(II), Ca, and potassium were negatively correlated to these OTUs (Fig. 9 and Fig. S7). Nearly half of the 50 most abundant OTUs (which accounted for >60% of the total sequences) were affiliated with putative phototrophs (Fig. 9). These observations highlight the significance of temperature and pH as important environmental determinants of microbial community composition and, specifically, the composition of anoxygenic phototrophs.

10.1128/mSystems.00498-19.7 FIG S7 Heatmap of Spearman correlation coefficient (Spearman’s rho) values between rarefied abundances of the top 50 most abundant OTUs classified at the class level and environmental variables. *P *values of <0.01 are indicated with an asterisk. Download FIG S7, PDF file, 0.3 MB. Copyright © 2019 Hamilton et al. 2019 Hamilton et al. This content is distributed under the terms of the Creative Commons Attribution 4.0 International license.

**FIG 10:**
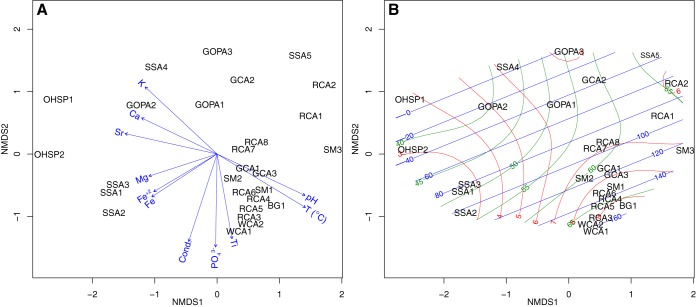
(A) Biplot of significant (*P < *0.01) environmental variables fitted on nonmetric multidimensional scaling (NMDS) based on Bray-Curtis dissimilarity between microbial communities across the 27 study sites. (B) NMDS with surface fitting of environmental variables: pH (red), temperature (green), and anoxygenic photosynthesis values (based on photoassimilation microcosms; blue).

Among the top 50 OTUs, many are most closely related to organisms capable of nitrogen fixation, including genera of *Cyanobacteria* and *Chlorobi* (Fig. 9 and Fig. S7). *Roseiflexus* populations have also been implicated in nitrogen fixation in alkaline hot springs (89, 90), although the complete genes for the synthesis and maturation of nitrogenase are not present in the genomes of these organisms. Regardless, OTUs with affiliated *Cyanobacteria*, *Chlorobi*, and *Chloroflexi* were more abundant in circumneutral to alkaline sites (Fig. 5 and Fig. S4), where the majority of biomass ^15^N values reflect biological nitrogen fixation (Fig. 7). These observations are consistent with N-limited springs selecting for the inclusion of organisms capable of N fixation. Molybdenum also had a statistically significant and strong influence on the top 50 OTUs (Fig. 9 and Fig. S7). However, Mo was not significant with respect to overall community structure (*P < *0.01) (Fig. 10A), despite being important for key processes in nitrogen, carbon, and sulfur cycling. The significant relationship between Mo and abundant OTUs could reflect a role for these populations in biological nitrogen fixation but may also be a result of the pH dependence on Mo concentration in Yellowstone hot springs. In alkaline hot springs, concentrations of NH_4_(T) tend to be low (<100 μM), due in part to the equilibration of aqueous NH_4_^+^_(aq)_ with NH_3(g)_ (pK 7.6 at 90°C) and the subsequent volatilization of NH_3(g)_ (81, 82). As a result of N limitation, alkaline terrestrial hot springs are thought to select for microbial populations with the capacity to fix N_2_ via the enzyme nitrogenase, which requires Mo (89, 91).

Nonmetric multidimensional scaling analysis (NMDS) indicated that, in addition to pH and temperature, potassium and conductivity were statistically significant environmental factors (*P < *0.01) related to the composition of microbial communities in our samples (Fig. 10A). In previous studies of YNP, pH and salinity also constrained phylogenetic diversity of *bchL/chlL* (5), and in the open ocean, salinity is the major environmental factor shaping aerobic anoxygenic phototroph taxonomy, while trophic status was also important (92). A similar role for salinity has been observed for global patterns of microbial diversity (93) as well as in freshwater and hypersaline lakes (94–97) and in YNP hot springs. In YNP, salinity is largely dependent on the availability and solubilities of salts in the subsurface flow paths (98). While salinity has been linked to the community structure of aerobic anoxygenic phototrophs in the open ocean and phototrophs in YNP hot springs, the combination of factors dictating their distribution (and activity) in geothermal springs, including pH, requires further study.

To examine the potential for biotic interactions to contribute to patterns in community and phototroph diversity, we carried out network analyses using CoNet app (99) as described above. However, we found that the analysis results were either oversimplistic, the effects due to the cooccurrence or the mutual exclusivity of OTUs was exaggerated, or given correlations between two OTUs obtained using different methods were contradictory to one another (data not shown). This, in combination with the fact that the metabolic lifestyles of many of the groups with phototrophs (i) are diverse, (ii) vary under different physiological conditions, or (iii) are not known inhibits meaningful interpretation of network connectivity based solely on correlating the cooccurrence or opposite occurrence (mutual exclusivity) of OTUs, even in combination with geochemical parameters.

Environmental fitting of pH, temperature, and rates of anoxygenic photosynthesis on the NMDS analysis using surface ordination clearly demonstrated the stratification of sites based on pH and temperature; however, we did not observe a clear relationship between the rates of anoxygenic photosynthesis (photoassimilation in the presence of DCMU) with either temperature or pH (Fig. 10B). Sulfide also was not significantly correlated to rates of anoxygenic photosynthesis or overall community structure (Fig. 10B). Several studies have indicated a role for sulfide in influencing the distribution of both oxygenic and anoxygenic phototrophs (3–5). Inhibition of photosystems by sulfide has been suggested to limit the distribution of oxygenic photosynthesis (4), while many anoxygenic phototrophs use sulfide as an electron donor (and employ reaction centers rather than photosystems). Previous studies have suggested sulfide is not an important substrate for phototrophy in YNP (5), and we recovered multiple OTUs of anoxygenic phototrophs that do not oxidize sulfide: *Roseiflexus* spp. (can grow photoheterotrophically under oxic conditions, including strains from YNP [84]), *Chloracidobacterium* (heterotroph that does not encode enzymes for the oxidation of sulfide [100]), and “*Ca.* Thermochlorobacteriaceae bacterium” GBChlB (an aerobic photoheterotroph that cannot use sulfide [57]).

While we did not observe a strong correlation between sulfide and rates of photoassimilation in the presence of DCMU (Fig. 4), we did recover OTUs affiliated with anoxygenic phototrophs that can oxidize sulfide and fix carbon. This, coupled to observed inorganic carbon assimilation in the presence of DCMU in circumneutral sites, suggests anoxygenic photosynthesis is active in these sites where H_2_S could be an electron donor. *Chloroflexus* can grow photoautotrophically with sulfide or hydrogen as an electron donor for carbon fixation via the 3HPB cycle, and *Chlorobi* can use sulfide as an electron donor for carbon fixation via the rTCA pathway. In general, circumneutral and alkaline sites exhibited higher rates of oxygenic and anoxygenic photosynthesis in higher temperature sites. Above a threshold of ∼1 μg C/g biomass C/h, there was an apparent correlation of increasing fractionation with increasing rates of anoxygenic photosynthesis (Fig. 8B). It is tempting to conclude the differences in Δ^13^C are due to the dominant carbon fixation pathways. For instance, carbon fixation via the CBB cycle in *Cyanobacteria* results in an isotopic fractionation of up to −25‰. In contrast, the 3HPB cycle (in autotrophic *Chloroflexi*) and the rTCA cycle (in autotrophic *Chlorobi*) typically generate a much smaller fractionation; however, these values can vary by temperature (51). Furthermore, an increase in anoxygenic photoautotrophic biomass production with smaller fractionation should lead to decreasing fractionation with increasing anoxygenic photosynthesis, the opposite of what was observed. Alternative explanations include variable incorporation of ^12^C-enriched allochthonous biomass via heterotrophy. Further work is needed to constrain potential effects of community composition and geochemical environments on associated putative carbon fractionation effects.

### Conclusions.

We expected pH, temperature, and sulfide to be key factors governing the distribution of physiologically diverse anoxygenic phototrophic taxa. While both temperature and pH play significant roles in structuring microbial communities in hot spring ecosystems, we observed a minimal role for sulfide. Our analyses identified pH as a key factor constraining both the physiology and taxonomy of anoxygenic phototrophs. We observed *Chloroflexi* populations across a wide range of temperature and pH, which is consistent with the metabolic and physiologic diversity of this phylum. Phototrophic *Chloroflexi* were constrained to sites with circumneutral to alkaline pH and higher temperature where they cooccur with phototrophic *Cyanobacteria*. Phototrophic *Proteobacteria* were rare in sites with circumneutral to alkaline pH despite their prevalence under these conditions in other systems. The factors that select for phototrophic *Cyanobacteria* and *Chloroflexi* under higher pH conditions while precluding phototrophic *Proteobacteria* warrant further testing. While we detected active anoxygenic phototrophs from pH 2.2 to 9.4 and in sites ranging in temperature from 31.5 to 71.0°C, we did not observe anoxygenic photoautotroph activity below pH 6. Instead, we suggest that eukaryotic oxygenic photosynthesis leads to the prevalence of aerobic anoxygenic photoheterotrophic taxa in acidic systems. Collectively, our observations suggest that, within the habitat range of phototrophs, pH is a key factor in constraining anoxygenic phototroph physiology and taxonomy, while the influence of other factors, including oxygen and sulfide, require further study. Abiotic and biotic factors likely interact to dictate the distribution of physiologically diverse anoxygenic phototrophs across physicochemical gradients, and the widespread distribution of phototrophy in YNP is consistent with a role for this metabolism in terrestrial geothermal springs throughout Earth’s history.

## MATERIALS AND METHODS

### Field site description.

Samples were collected from geothermal features in Yellowstone National Park in October of 2015, June of 2016, and July of 2017. Geothermal features were targeted to capture a range of temperatures and pH as well as other geochemical parameters, including sulfide (see Table S1 in the supplemental material). Samples, including mats, filaments, floc, and sediments (Fig. S1 shows pictures of representative pictures of mats, filaments, and floc examined in the present study). A total of 27 samples were collected from five hydrothermal areas: the Sylvan Springs Area (SSA, *n* = 5) and the Geyser Creek Area (GCA, *n* = 3) in the Gibbon Geyser Basin (GGB); Sentinel Meadows (SM, *n* = 3), Boulder Geyser (BG, *n* = 1), and the White Creek Area (WCA, *n* = 2) in the Lower Geyser Basin (LGB); the Rabbit Creek Area (RCA, *n* = 8) in the Midway Geyser Basin (MGB); the Greater Obsidian Pool Area (GOPA, *n* = 3) in the Mud Volcano Area (MVA); and the One Hundred Springs Plain (OHSP, *n* = 2) area of Norris Geyser Basin (NGB). GPS coordinates of sample sites are provided in Table S1.

### Sample collection and geochemistry.

Samples for nucleic acid extraction (*n* = 3 for each site) and C and N content and δ^13^C and δ^15^N determination (*n* = 1 for each site) were collected using flame-sterilized spatulas or forceps. Samples were placed in sterile 2.0-ml vials and immediately frozen on dry ice and stored at –80°C until nucleic acid extraction or processing for C, N, δ^13^C, and δ^15^N analysis.

Temperature and pH were measured onsite using a WTW 330i meter and probe (Xylem Analytics, Weilheim, Germany), and conductivity was measured with a YSI 30 conductivity meter and probe (YSI Inc., Yellow Springs, OH, USA). Sulfide, Fe^2+^, and dissolved silica were measured onsite using a DR1900 portable spectrophotometer (Hach Company, Loveland, CO). Water samples were filtered through 0.2-μm polyethersulfone syringe filters (VWR International, Radnor, PA, USA) and analyzed for cation concentration (Na, K, Ca, and Mg), anion concentration (Cl^−^ and SO_4_^2−^), trace element concentration (P, Mn, Fe, As, and Mo), dissolved inorganic carbon (DIC) concentration and δ^13^C value, and dissolved organic carbon concentration and δ^13^C value, as described previously (101–103). Field blanks comprised of filtered 18.2 MΩ/cm deionized water, transported to the field in 1-liter Nalgene bottles (acid washed as described above), were collected onsite using the equipment and techniques described above.

For ion chromatography analysis of anions, water was filtered into 15-ml centrifuge tubes (presoaked in 18.2 MΩ/cm deionized [DI] water) and stored at 4°C until analysis. Major anions were determined using a Dionex ICS2500 ion chromatography (IC) system (Dionex, Sunnyvale, CA, USA) by the Earth and Environmental Systems Institute (EESI) (The Pennsylvania State University, University Park, PA, USA), the STAR Lab at the Ohio State University, or the Analytical Geochemistry Laboratory in the Department of Earth Sciences at the University of Minnesota as described previously.

Samples for cation analysis were filtered into acid-washed 15-ml centrifuge tubes (three-day soak in 10% TraceMetal-grade HNO_3_ [Fisher Scientific, Hampton, NH, USA] followed by triple rinsing with 18.2 MΩ/cm DI water) and immediately acidified with 400 μl of concentrated OmniTrace Ultra concentrated nitric acid (EMD Millipore, Billerica, MA, USA). Samples were stored at 4°C until analysis. Analysis of cations was conducted via a Thermo X-Series II quadrupole collision cell technology-enabled inductively coupled plasma mass spectrometer (Thermo Fisher Scientific, Waltham, MA, USA) by the EESI at the Pennsylvania State University, the STAR Lab at the Ohio State University, or the Analytical Geochemistry Laboratory in the Department of Earth Sciences at the University of Minnesota.

Four-milliliter samples for DIC concentration analysis were filtered into Labco Exetainers (Labco Limited, Lampeter, UK) preflushed with helium. Samples were stored cap-side down at 4°C until analysis. DIC concentrations and δ^13^C were determined by the Stable Isotope Facility at the University of California, Davis, using a GasBench II system interfaced to a Delta V Plus isotope ratio mass spectrometer (IR-MS) (Thermo Scientific, Bremen, Germany). Raw delta values were converted to final values using laboratory standards (lithium carbonate, δ^13^C = −46.6‰; deep seawater, δ^13^C = +0.8‰) calibrated against standards NBS-19 and L-SVEC.

### Stable isotope signals of biomass carbon and nitrogen.

C and N stable isotope signals of biomass were determined via a Costech Instruments elemental analyzer (EA) periphery connected to a Thermo Scientific Delta V Advantage IR-MS at the UC Davis Stable Isotope Facility. Microcosm samples were thawed, and biomass was rinsed with 1 M HCl to remove any extra ^13^C-labeled DIC, triple rinsed with 18.2 MΩ/cm deionized water, and then dried (60°C for 3 days). Natural abundance samples were not treated with acid. Samples were ground/homogenized with a cleaned mortar and pestle (ground with ethanol silica slurry, triple rinsed with 18.2 MΩ/cm deionized water, and dried), weighed, and placed into tin boats, sealed, and submitted to the UC Davis Stable Isotope Facility for analyses.

All isotopic data are reported as isotope ratios, relative to standards of known value, using the equation [(isotope ratio of sample)/(isotope ratio of standard)^−1^] × 1,000, expressed in delta notation (δ) and reported as per mil (‰). Carbon isotopic values are reported as isotopic ratios of ^13^C to ^12^C from the equation [(^13^C/^12^C of sample)/(^13^C/^12^C of standard)^−1^] × 1,000 and expressed in delta notation (δ^13^C). δ^13^C values are reported using the Vienna Pee Dee Belemnite (VPDB) standard. The difference between DIC δ^13^C values and biomass δ^13^C values is expressed as Δ^13^C and represents the fractionation of ^13^C from the source inorganic carbon (DIC δ^13^C) and biomass δ^13^C values. Nitrogen isotopic values are reported as isotopic ratios of ^15^N to ^14^N from [(^15^N/^14^N sample)/(^15^N/^14^N standard)^−1^] × 1,000, expressed in delta notation (δ^15^N). The standard for reporting nitrogen isotopic values is atmospheric N_2_.

To minimize cross contamination of natural abundance samples with incubation assays, natural abundance samples were analyzed prior to incubation assays and laboratory processing, and weighing equipment was cleaned with 80% ethanol between each sample. Standard checks and blanks were included in each run to check for memory effects or cross contamination of samples, with none detected. C and N and stable isotope analyses are provided in Table S1.

### CO_2_ assimilation (microcosms).

The potential for inorganic carbon uptake *in situ* was assessed using microcosms through the addition of NaH^13^CO_3_. Microcosms were performed at the time of sample collection (described above) in October of 2015, June of 2016, and July of 2017. Samples were collected as described above, and ∼300 mg was placed into precombusted (12 h, 450°C) serum vials. When appropriate, the structure of mats or filaments was maintained. Care was taken to minimize exposure of samples to oxygen and maintain *in situ* temperature: samples are immediately capped upon collection and stored in water collected from the sample collection site. Transfer to serum vials occurs immediately after sample collection, followed by addition of spring water (10 ml) from the collection site and capping of the serum vials with gas-tight black butyl rubber septa. All amendments were then added by gas-tight syringe injection.

Microcosms were initiated by addition of NaH^13^CO_3_ (100 μM final concentration) (Cambridge Isotope Laboratories, Inc., Andover, MA, USA). All microcosms were performed in triplicate and incubated at spring temperature for ∼2 h. Microcosms were performed between noon and 4 p.m. under full or partial sun. We assessed the potential for photoautotrophic (light) and chemoautotrophic (dark) NaH^13^CO_3_ uptake. In addition, the contribution of photosystem II (PSII)-independent anoxygenic photoassimilation of CO_2_ was determined in assays amended to a final concentration of 10 μM DCMU [PSII inhibitor; 3-(3,4-dichlorphenyl)-1,1-dimethylurea] (Sigma-Aldrich, St. Louis, MO, USA). Assays were stopped by flash freezing vials on dry ice. Incubation duration times were recorded in the field. All vials were stored at –80°C until processed (described above). Reported values of ^13^C-labeled DIC uptake (carbon fixation rates) reflect the difference in uptake between the biomass in the assays that received NaH^13^CO_3_ and the natural abundance biomass samples. Using the organic carbon content, the uptake rate was calculated from the total micrograms of C taken up divided by the grams of organic C per gram of sediment, and that was divided by the number of hours of incubation (typically ∼2 h). Carbon assimilation rates are provided in Table S2. For comparisons between mean ^13^C uptake rates, one-way analysis of variance (ANOVA) followed by *post hoc* pairwise comparisons between treatments was conducted using Tukey’s honest significant difference (HSD) within the R software package (R, version 3.3.2). Mean rates with *P* values of <0.05 were considered significantly different (Table S2). Our microcosm approach could underestimate anoxygenic photosynthesis in several ways, including inhibition of activity due to the introduction of oxygen during microcosm setup, substrate limitation due to consumption during the incubation period, and lack of DCMU penetration into mats and filaments. We employed small sample sizes (100 s of mg), performed short incubations (∼2 h), and did not anticipate lack of DCMU penetration into samples; DCMU is a specific PSII inhibitor that acts at very low concentrations, but the effects are also reversible and can be removed by washing (104).

### Nucleic acid extraction.

Total DNA was extracted from triplicate ∼250-mg samples using a DNeasy PowerSoil kit (Qiagen, Carlsbad, CA, USA) according to the manufacturer’s instructions. Total RNA was extracted from triplicate ∼250-mg samples using a RNeasy power biofilm kit (Qiagen, Carlsbad, CA, USA), which includes a DNase treatment, according to the manufacturer’s instructions. A second DNase treatment was performed according to the Joint Genome Institute (JGI) Sequencing Technology DNase treatment protocol (https://jgi.doe.gov/user-programs/pmo-overview/protocols-sample-preparation-information). Briefly, RNA was incubated at 37°C in the presence of DNase I and DNase I reaction buffer (Ambion, Thermo Fisher Scientific) for 30 min, followed by a cleanup step using the RNeasy MinElute cleanup kit (Qiagen, Carlsbad, CA, USA), modified according to the JGI protocol. Concentrations of DNA and RNA were determined using a Qubit 3.0 fluorometer (Invitrogen, Burlington, ON, Canada), and equal volumes of each extraction were pooled prior to sequencing or qRT-PCR analyses. DNA was extracted from the filter used for the field blank water sample as a negative DNA extraction control. No DNA was detected in any of the filter controls, and sequencing failed to generate amplicons (see below for amplicon sequencing details). RNA extracts were screened for the presence of contaminating genomic DNA by performing a PCR using ∼1 ng of RNA as the template and bacterial 16S rRNA gene primers 1100F and 1492R as described previously (105).

### Sequence analysis.

Amplicons were sequenced using MiSeq Illumina 2× 300-bp chemistry at the University of Minnesota Genomics Center (UMGC). We targeted the V4 hypervariable region of bacterial and archaeal 16S small subunit rRNA gene using the modified primers 515F (5′-TCGTCGGCAGCGTCAGATGTGTATAAGAGACAGGTGCCAGCMGCCGCGGTAA-3′) and 806R (5′-GTCTCGTGGGCTCGGAGATGTGTATAAGAGACAGGGACTACHVGGGTWTCTAAT-3′). Amplicon libraries were created by UMGC by following their improved protocol for library preparation, which enables detection of taxonomic groups that often go undetected with existing methods (106). Each sample was sequenced once. Postsequence processing was performed within the mothur (ver. 1.37.6) sequence analysis platform (107) following MiSeq SOP (108) as described previously (101). Briefly, read pairs were assembled and resulting contigs with ambiguous bases were removed. Contigs were trimmed to include only the overlapping regions, and unique sequences were aligned against a SILVA-based reference alignment and classified using a Bayesian classifier within mothur against the Silva (v132) reference taxonomy. Chimeras were identified in the aligned sequences using UCHIME (119) with the Silva SEED database (v132) and removed from further analyses. Singletons were removed using the “*remove.rare*” function within mothur.

Following removal of chimeras and singletons, sequences were binned into operational taxonomic units (OTUs) based on a sequence similarity of 97.0% for archaea and bacteria. OTUs assigned to mitochondria or chloroplast were removed and not included in the rest of the analyses. OTUs with unassigned taxonomy by mothur were further refined using BLASTN and GreenGenes databases using BLCA and assigned taxonomy when possible (109). Following quality control, merging of contigs, and removal of chimeras and singletons, we recovered 930,251 16S rRNA gene sequences. The libraries ranged in size from 6,345 to 117,014 total sequences. At a sequence identity of 97%, we recovered 12,748 total OTUs, 915 OTUs affiliated with archaea, and 11,833 OTUs affiliated with bacteria. The phototrophic taxa were grouped by phylum *Chloroflexi*, *Cyanobacteria*, and *Gemmatimonadetes*; class *Alphaproteobacteria*, *Betaproteobacteria*, *Gammaproteobacteria*, and *Chlorobia*; and family *Heliobacteria*. We recognize that not all representatives from these groups are known phototrophs and have provided higher taxonomic resolution in the supplemental information.

### Rank abundance plots.

Rank abundance plots were generated in R, version 3.4.3 (110), using the packages ggplot2 (111), tidyverse (112), and dplyr (113). OTU tables and taxonomy files generated from mothur were imported into R. OTUs were binned according to location pH or location temperature and pooled, generating three bins per geochemical parameter, and absolute abundance of each OTU within that bin was calculated. OTUs were ranked by decreasing absolute abundance in a given bin, and the top 50 OTUs were plotted by decreasing absolute abundance.

### Statistical analysis.

All statistical analyses were carried out in R, version 3.4.3 (110). Rarefaction data and relative abundance were plotted using ggplot2 (111). For analysis of alpha diversity, each sample was rarefied to a depth of 6,345 (the total reads from the SSA2 site; Fig. S2). At this sequencing depth, there are several samples (e.g., RCA8 and SM3) that have not yet plateaued despite more than 20,000 reads for each library; thus, we may underestimate the diversity. Alpha diversity metrics were calculated using RTK (114). The median values were obtained from 1,000 pseudoreplicates. Environmental fitting and nonmetric multidimensional scaling (NMDS) ordination analyses were carried out with vegan using “*metaMDS”* and “*envfit*,*”* respectively (115) (environmental parameters with a *P *value of *<*0.01 were considered significant). Linear regression models between relative abundance of OTUs and pH or temperature were built using the “*stat_fit”* method in ggplot2. Spearman correlation *R* values were plotted using ComplexHeatmap (116). Relative abundance of OTUs was determined by calculating the count of the OTU divided by the total OTUs of a given site. Relative abundance within pH and temperature bins was determined by totaling the count for an OTU across sites within a bin and dividing by the total count of all OTUs within the bin. Network analyses was performed using CoNet (99). Briefly, Spearman/Kendall/Pearson correlation, scaled variance of log ratios (>0.8 or <−0.8), and Fisher’s Z (*P* < 0.05, Bonferroni corrected) were calculated and visualized in Cytoscape (117). To evaluate the association between ^13^C assimilation rate and environmental parameters (pH, temperature, and sulfide), a Spearman rank correlation test was conducted using the base R package. ^13^C assimilation rates were plotted across pH, temperature, and sulfide space, and Spearman rank correlation lines were drawn and plotted with correlation coefficients (*R*) and *P* values using the ggpubr package. The association between *bchY* transcript abundance and environmental parameters (pH, temperature, and sulfide) were calculated and plotted using the same methods.

10.1128/mSystems.00498-19.2 FIG S2 Rarefaction output from mothur for OTUs observed at the 97% OTU level are plotted for all of the study sites. The dashed blue line (6,345 reads) indicates the lowest sample size for the site SSA2. Site and area designations are provided in Table S1. Download FIG S2, PDF file, 0.3 MB. Copyright © 2019 Hamilton et al. 2019 Hamilton et al. This content is distributed under the terms of the Creative Commons Attribution 4.0 International license.

### *bchL* and *bchY* distribution.

The occurrence of *bchY* was assayed by amplifying a 500-bp fragment of *bchY* using *bchY*_fwd (5′-CCNCARACNATGTGYCCNGCNTTYGG-3′ and *bchY*_rev (5′-GGRTCNRCNGGRAANATYTCNCC-3′) as described previously (5, 43). The occurrence of *chlL/bchL* was assayed by amplifying a 390-bp fragment of *chlL/bchL* using *chlLbchL-*54F (5′-CARATYGGHTGYGAYCCNAARCAYGA-3′) and chlL*bchL-167R (5′-AAYGRYTTYGAYDSBWTHTTYGC-3′), as described previously (5, 43). *bchY* encodes a component of chlorophyllide oxidoreductase, which reduces the B-ring of Chlide *a* during pigment biosynthesis of BChl *a*, *b*, and *g* in anoxygenic phototrophs (118). Primers to amplify *bchY* have been employed as a proxy to characterize the distribution and diversity of anoxygenic phototrophs (*Proteobacteria*, *Chlorobi*, *Chloroflexi*, *Acidobacteria*, and *Firmicutes*) (5, 42, 43). *bchY* is only present in anoxygenic phototrophs, while all known bacterial phototrophs encode *chlL/bchL* (41, 44, 45).

### qRT-PCR.

The abundance of *bchY* transcripts was assayed by amplifying a 500-bp fragment of *bchY* using *bchY*_fwd and *bchY_*rev as described previously (5, 43). qRT-PCR was performed using the Power SYBR green RNA-to-CT one-step kit according to the manufacturer’s protocol. Reactions were assayed using a StepOne Plus real-time PCR system. Reactions were performed in triplicate with 5 ng of total RNA quantified as described above, with 200 nM forward and reverse primer in a final reaction volume of 20 μl. Control reaction mixtures contained either no reverse transcriptase or no template RNA.

### Data availability.

All sequence data, including raw reads with quality scores for this study, have been deposited in the NCBI Sequence Read Archive (SRA) database under the BioProject number PRJNA513338. Library designations are provided in Table S1.
