# Supplementary figures and images for: Anoxygenic Phototrophs Span Geochemical Gradients and Diverse Morphologies in Terrestrial Geothermal Springs
Source: mSystems. 2019 Nov 5;4(6):e00498-19. doi: 10.1128/mSystems.00498-19 (PMC6832021; doi:10.1128/mSystems.00498-19)

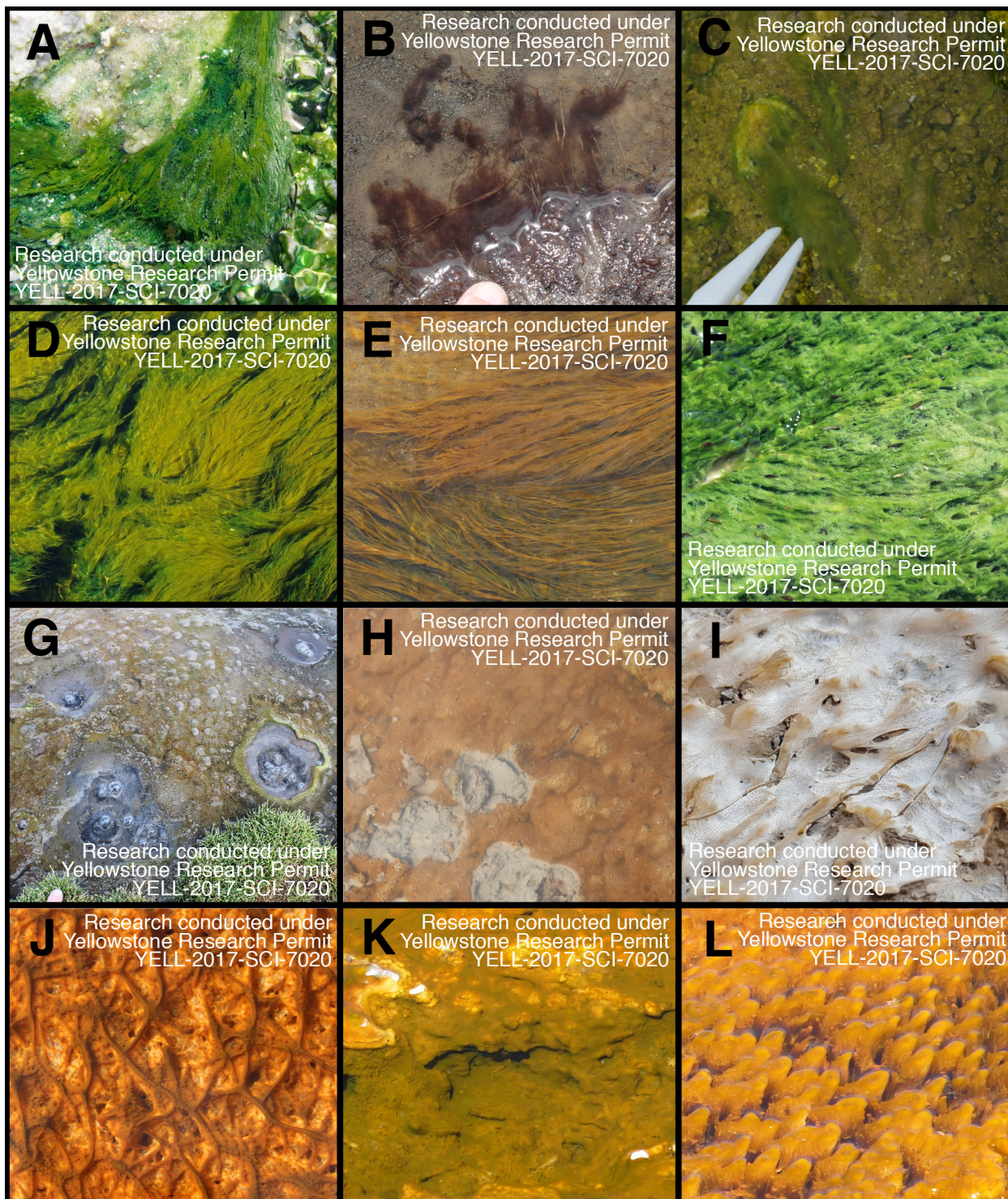

Supplement: FIG S1 [file mSystems.00498-19-sf001.pdf]

**A**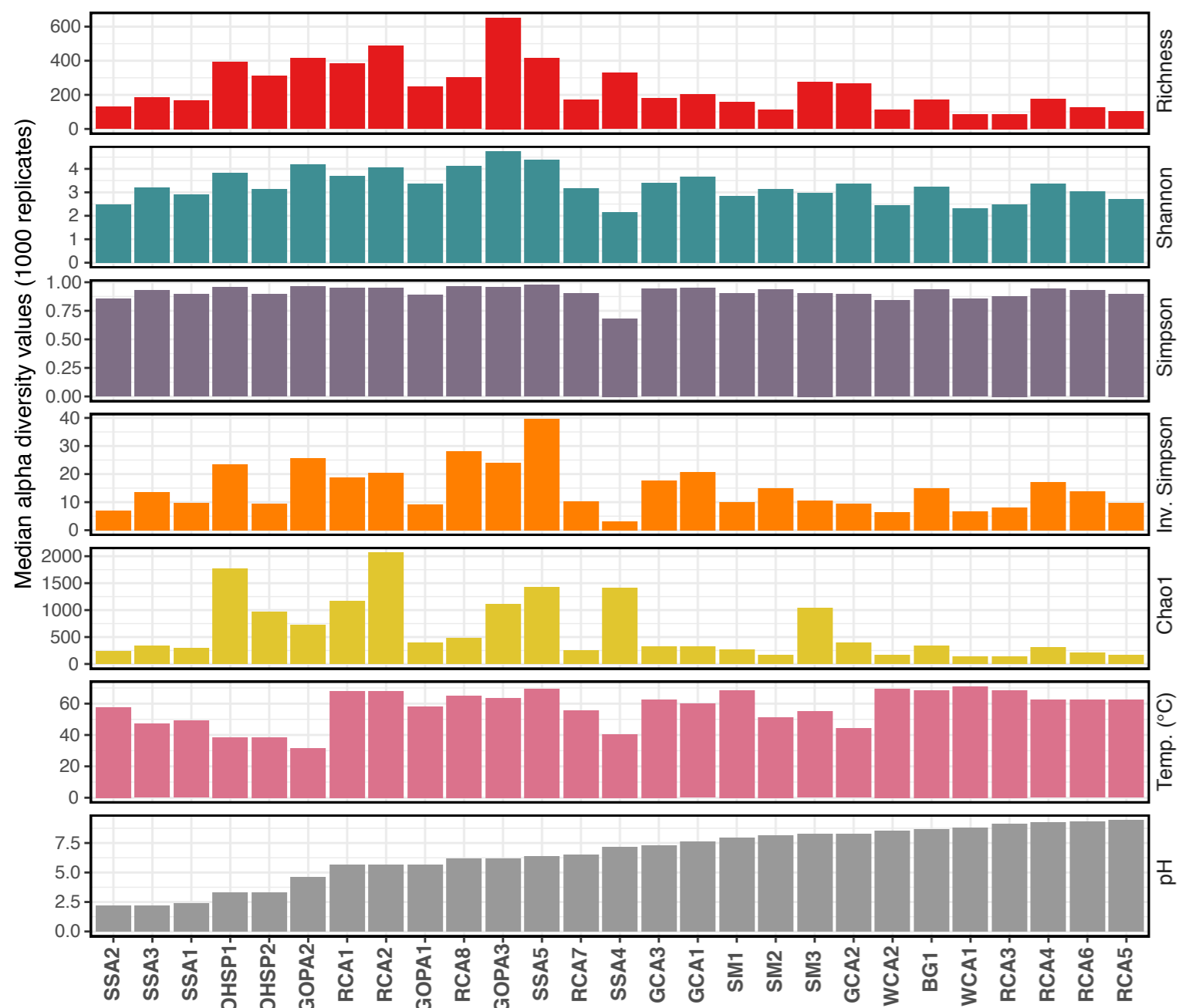**B**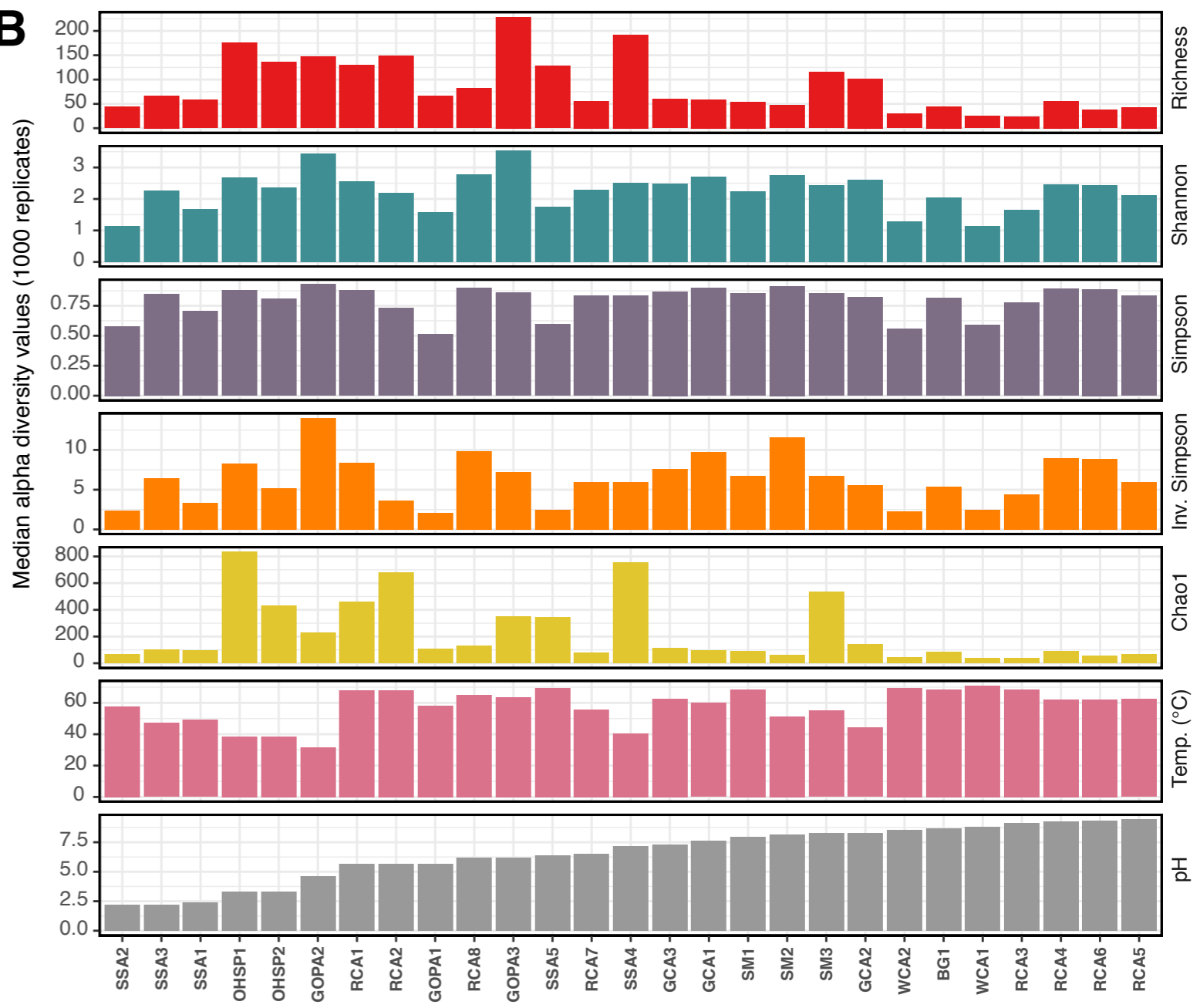

Supplement: FIG S3 [file mSystems.00498-19-sf003.pdf]

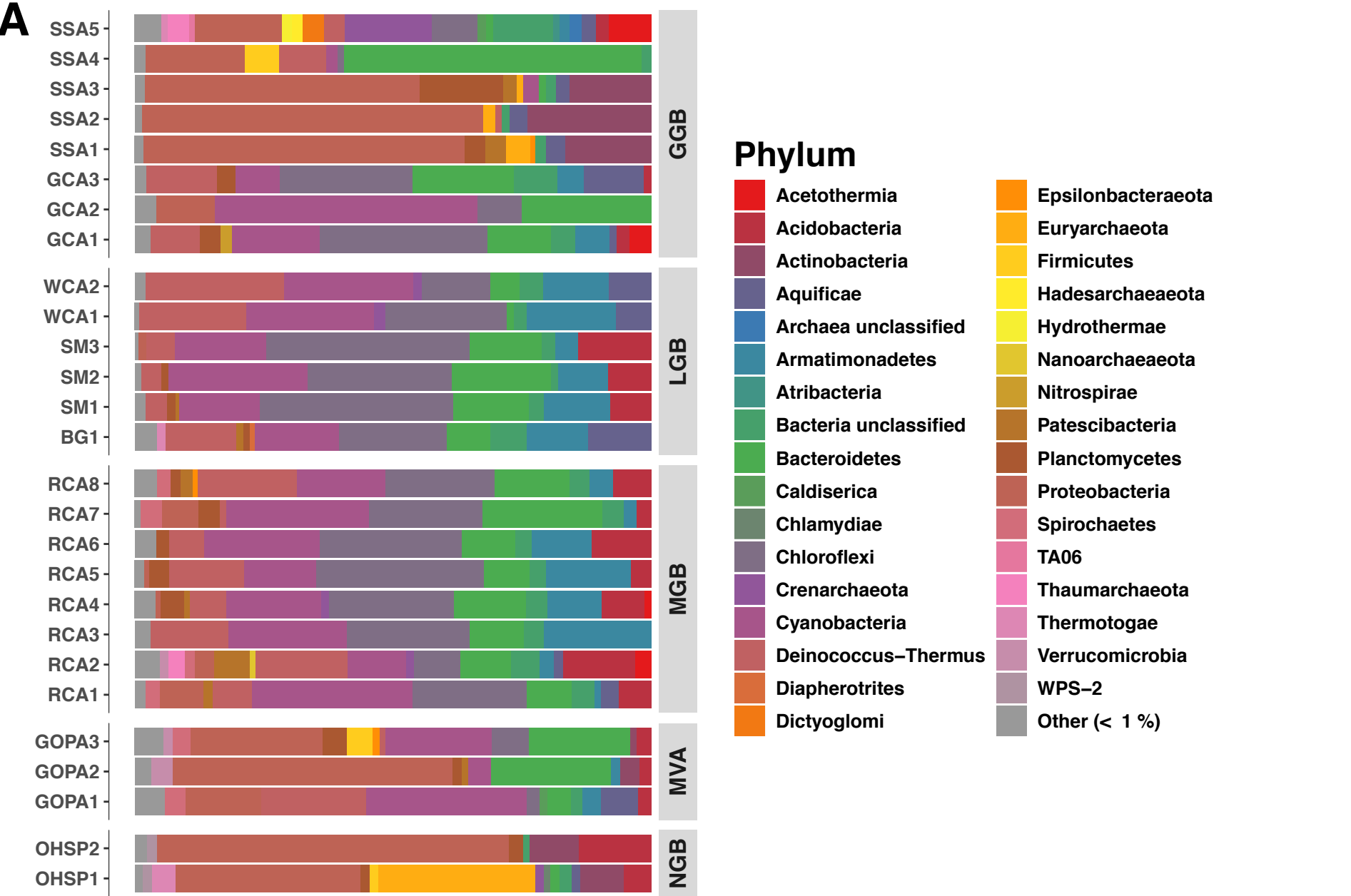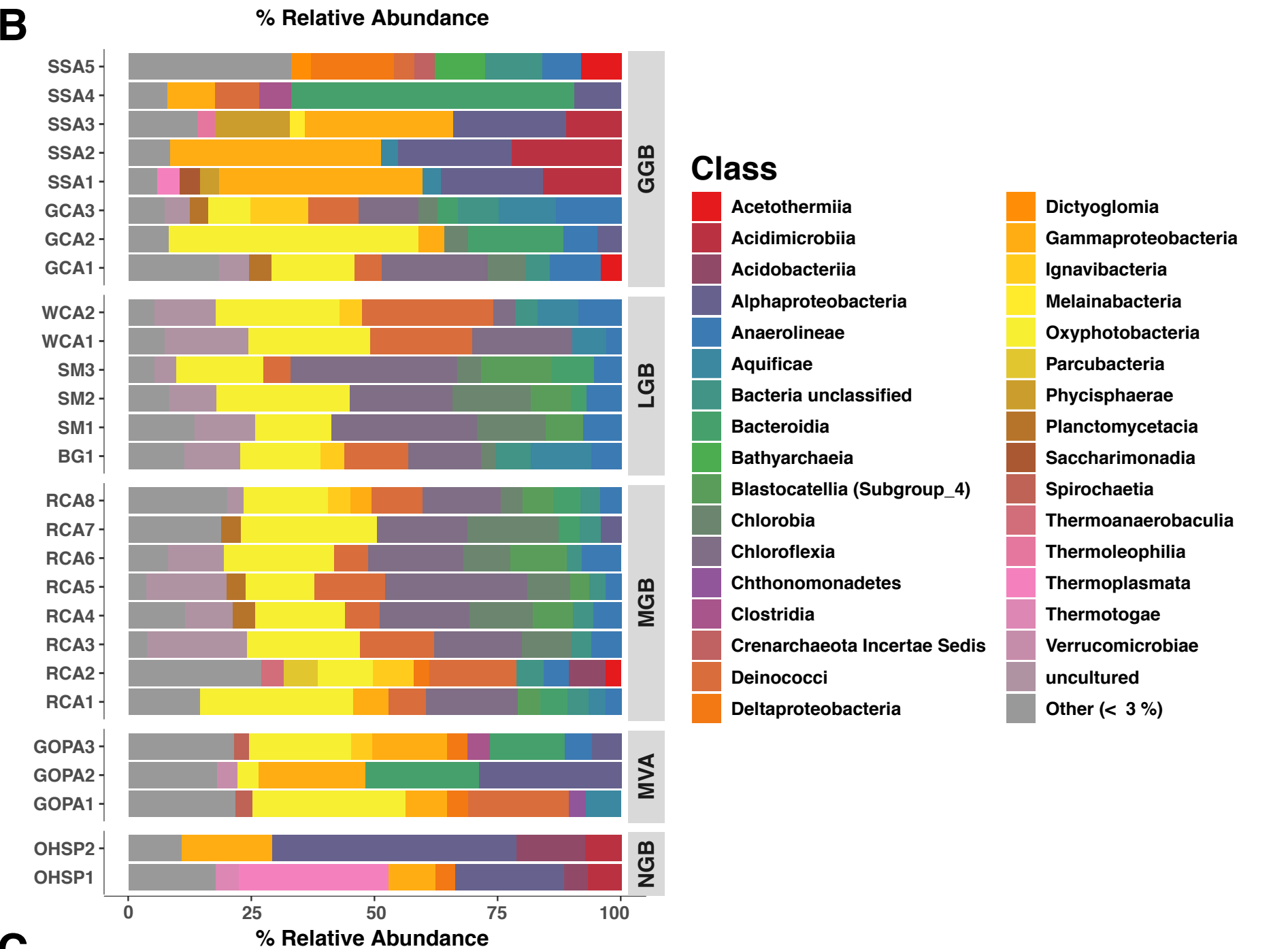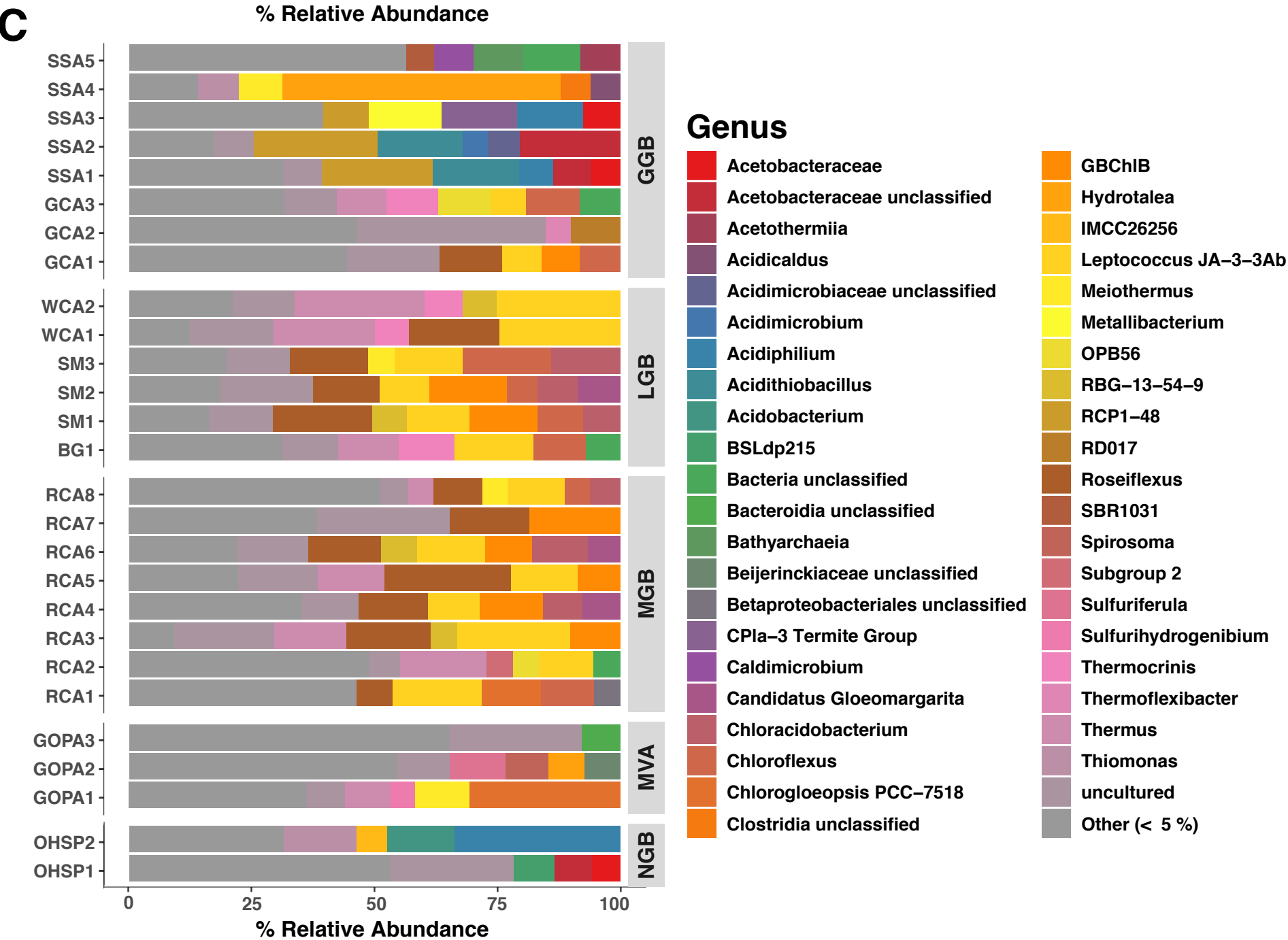

Supplement: FIG S4 [file mSystems.00498-19-sf004.pdf]

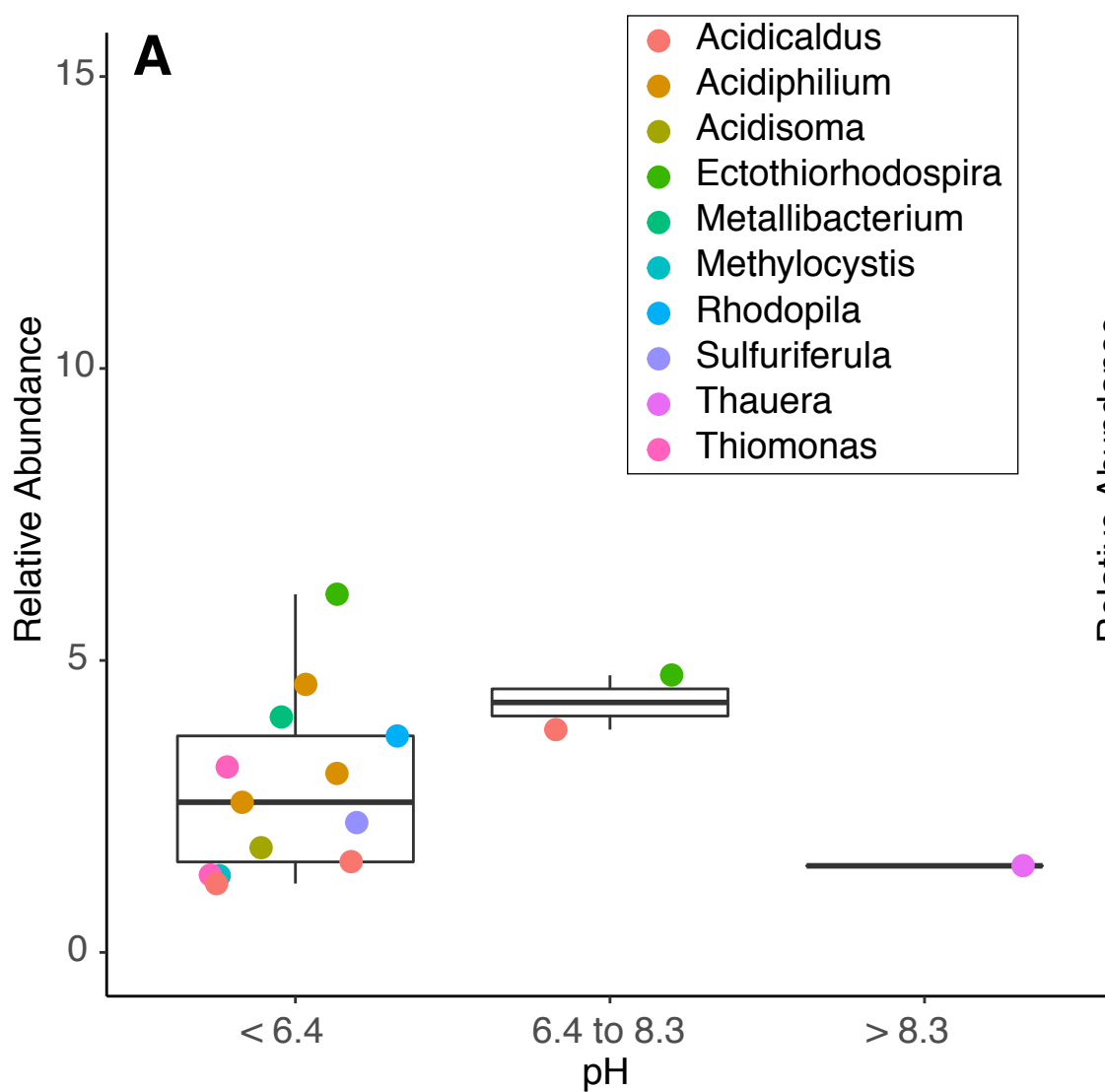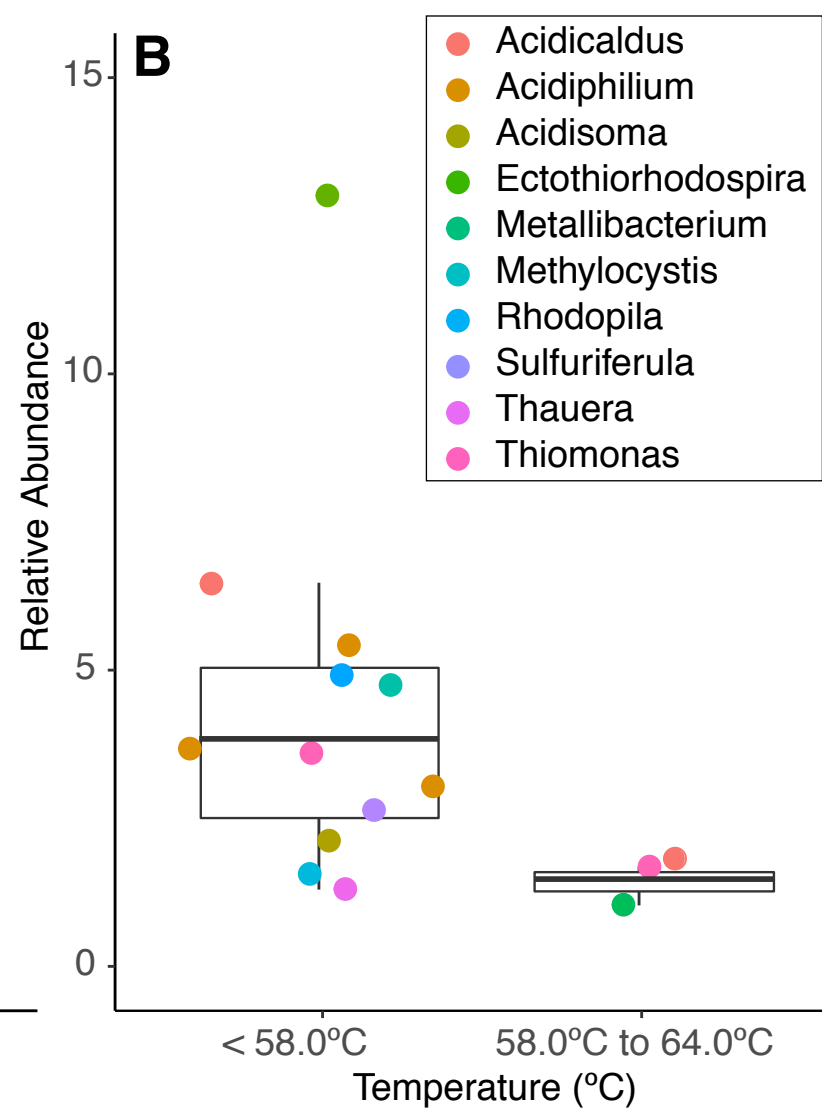

Supplement: FIG S5 [file mSystems.00498-19-sf005.pdf]

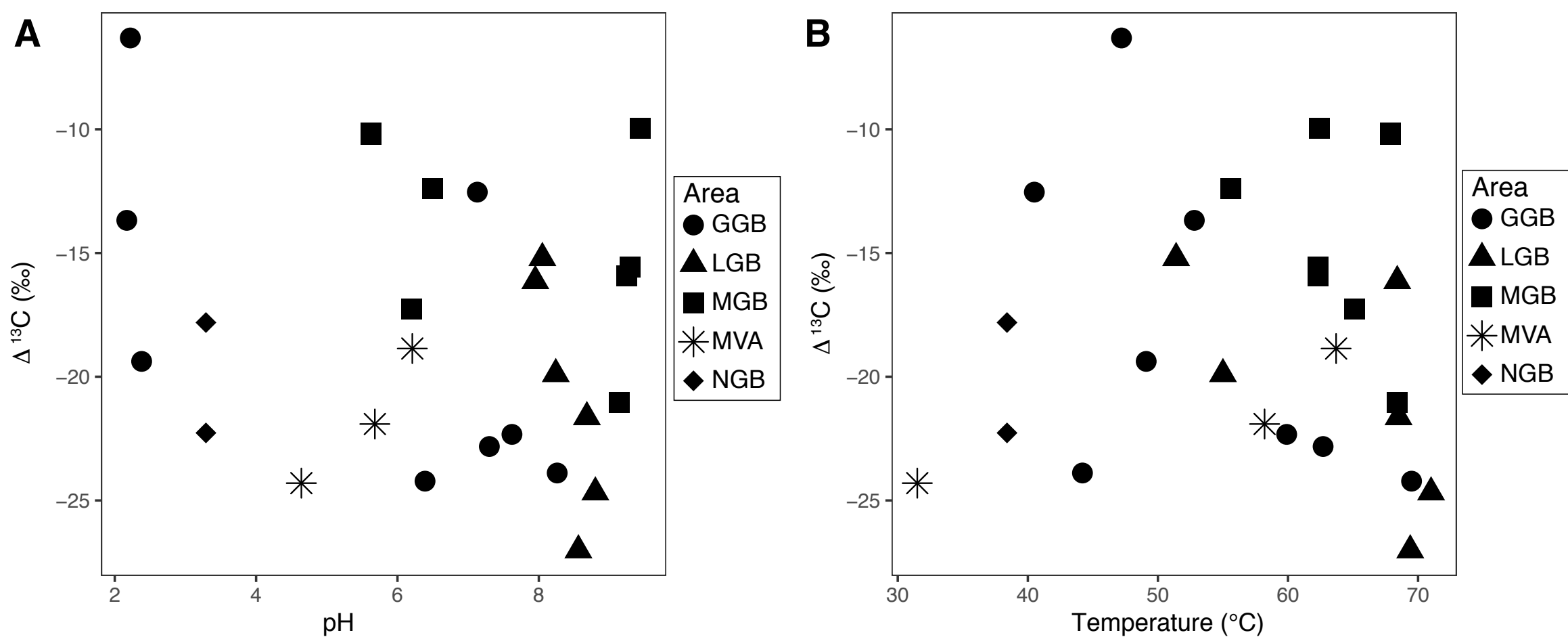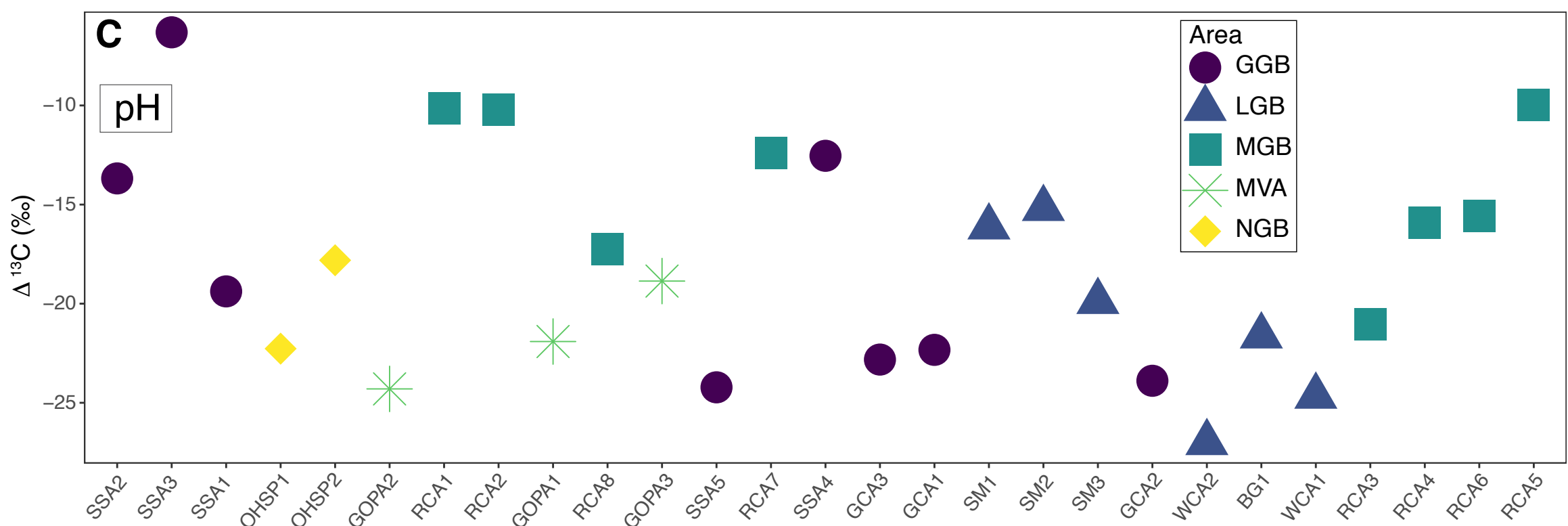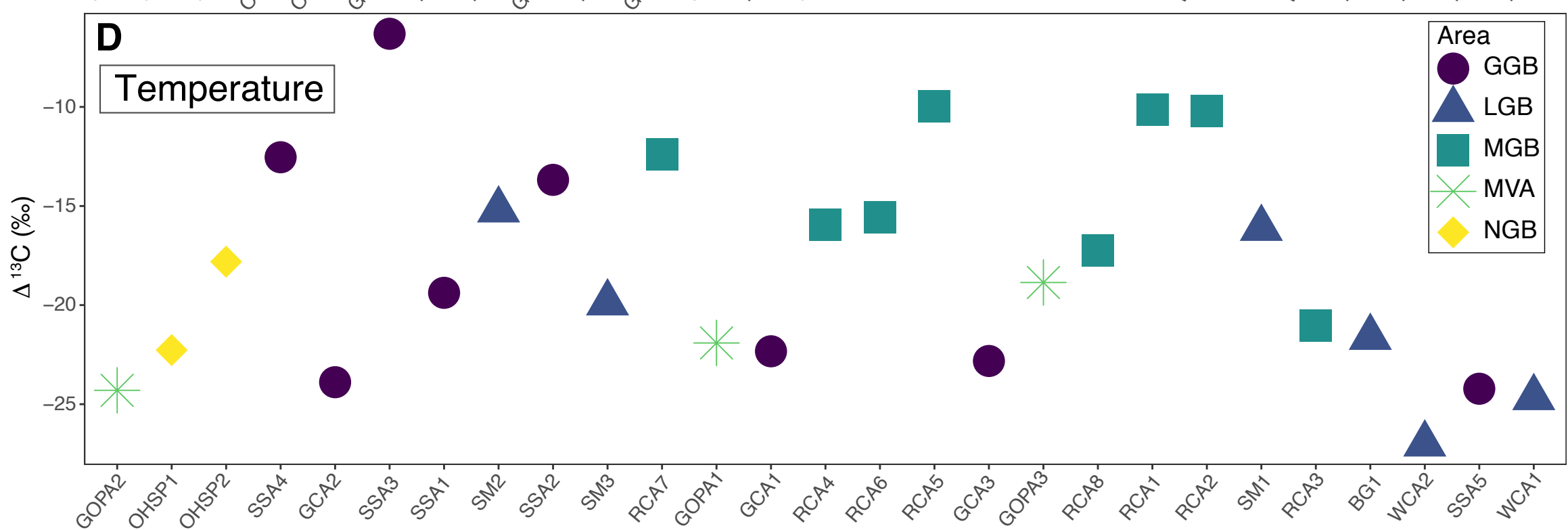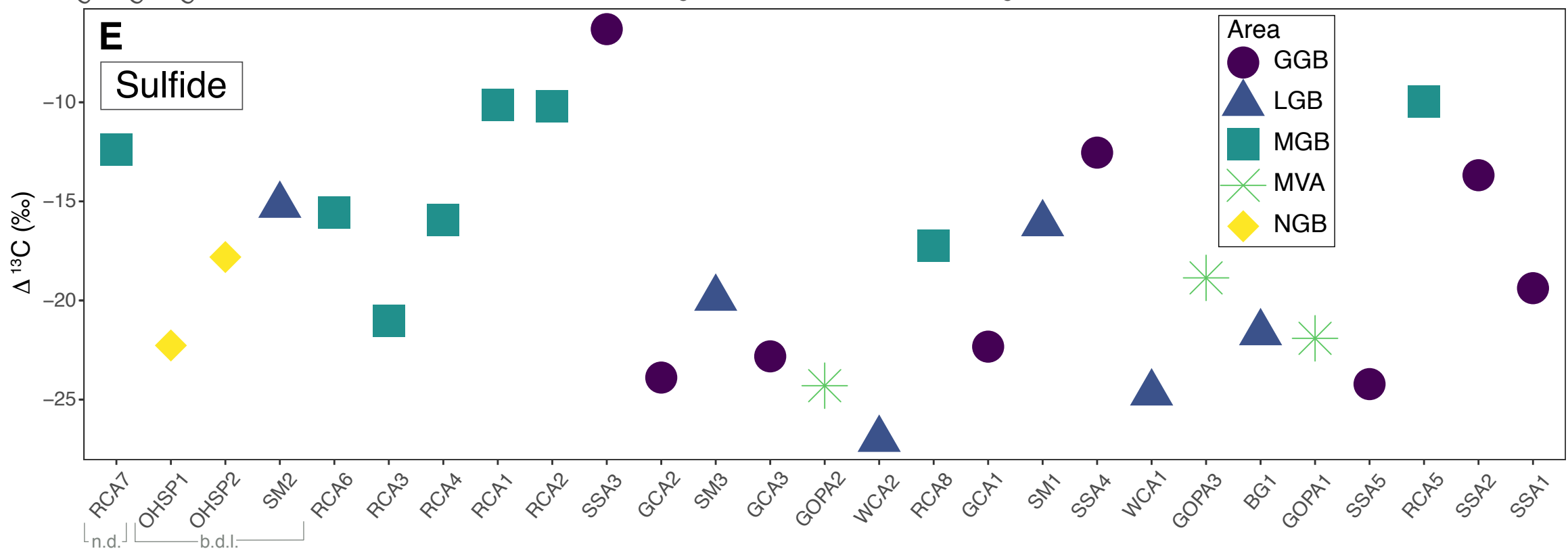

Supplement: FIG S6 [file mSystems.00498-19-sf006.pdf]

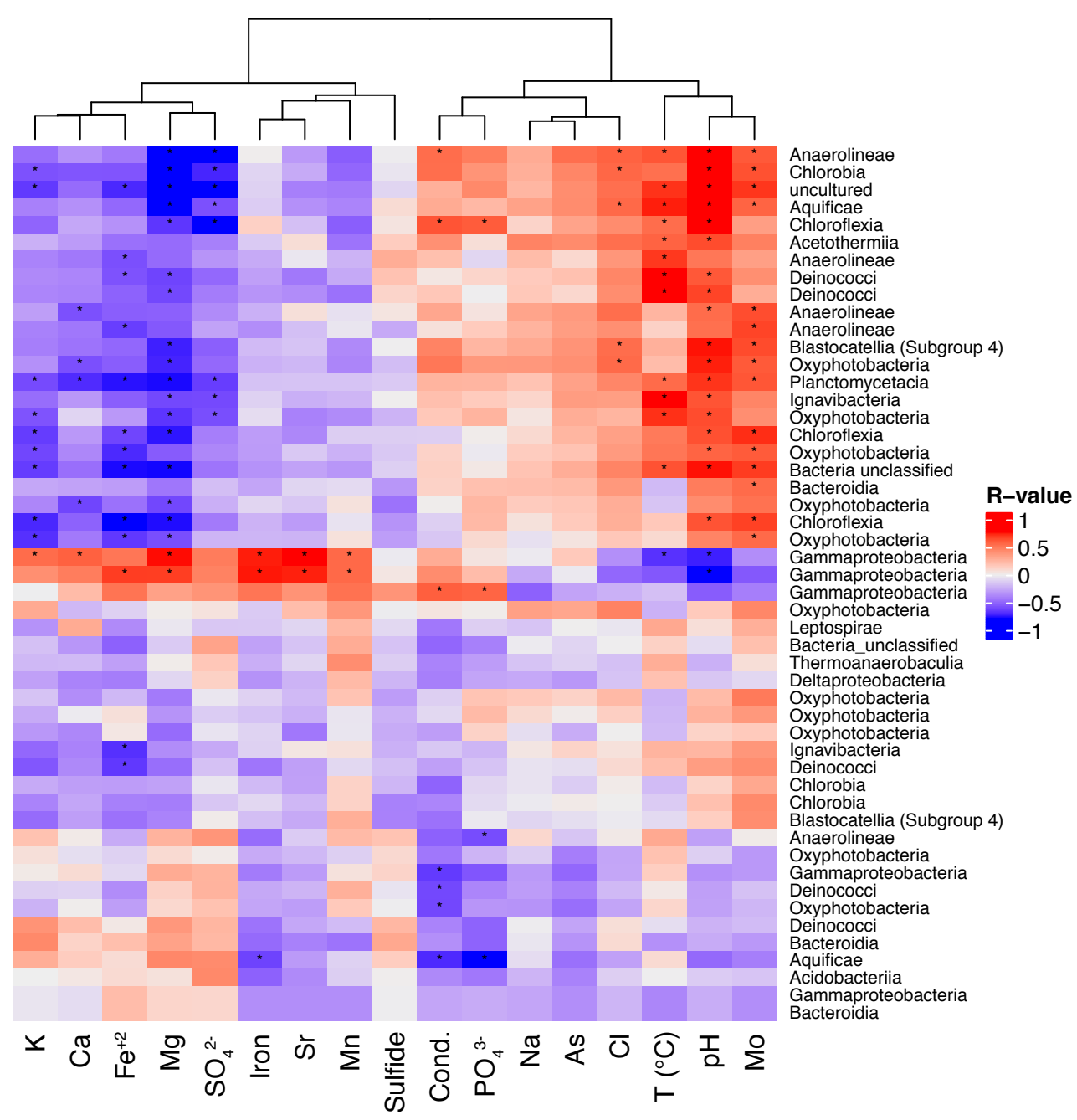

Supplement: FIG S7 [file mSystems.00498-19-sf007.pdf]

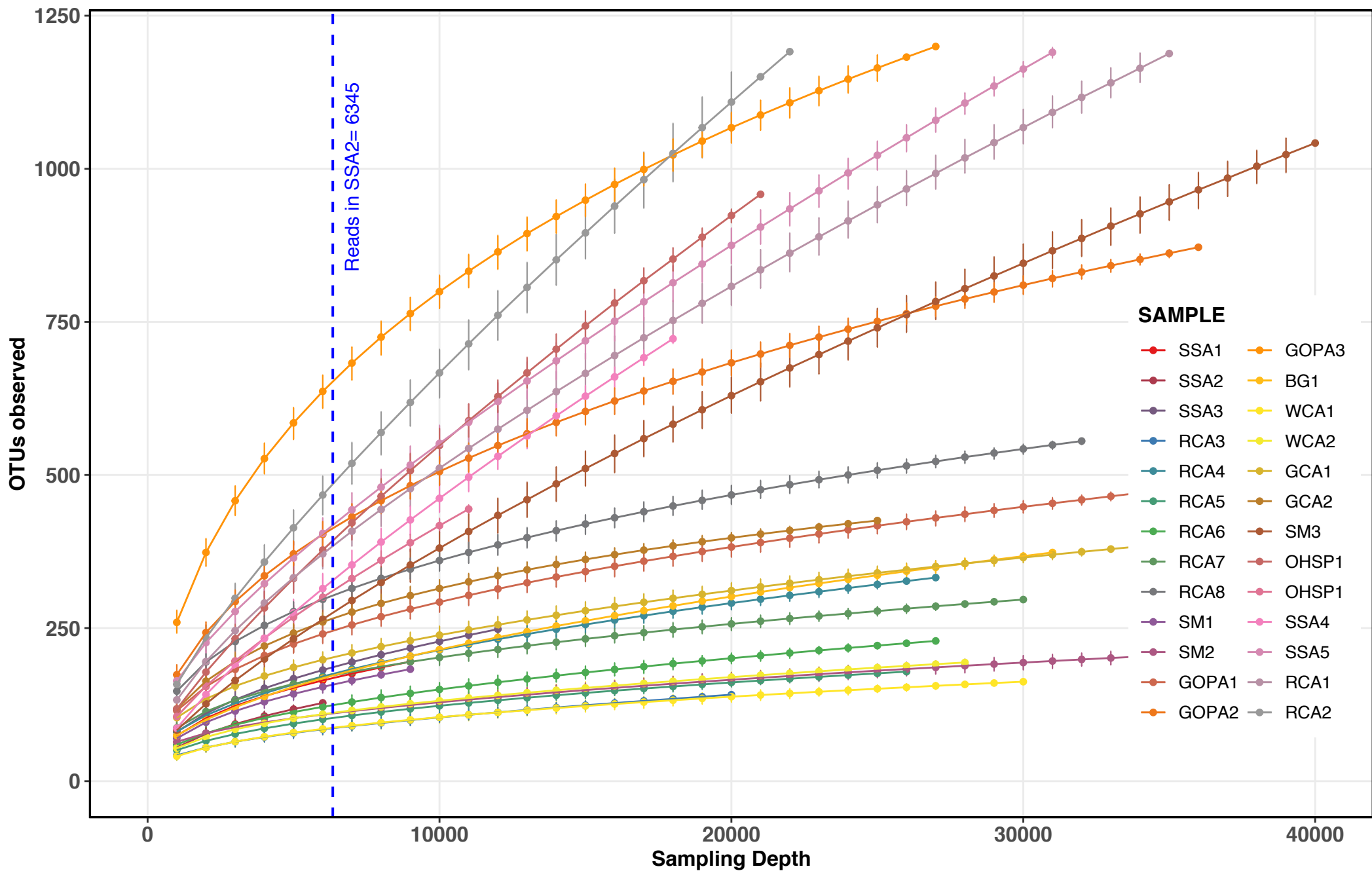

Supplement: FIG S2 [file mSystems.00498-19-sf002.pdf]
